# A conserved terpene cyclase gene in *Sanghuangporus* for abscisic acid-related sesquiterpenoid biosynthesis

**DOI:** 10.1186/s12864-025-11542-9

**Published:** 2025-04-15

**Authors:** Yoonhee Cho, Chang Wan Seo, Hyeonjae Cho, Yeongwoon Jin, Abel Severin Lupala, Sang Hee Shim, Young Woon Lim

**Affiliations:** 1 School of Biological Sciences and Institute of Biodiversity, Seoul National University, Seoul, 08826 Republic of Korea; 2 College of Pharmacy, Natural Products Research Institute, Seoul National University, Seoul, 08826 Republic of Korea; 3 Department of Microbiology, Parasitology and Biotechnology, Sokoine University of Agriculture, P.O. Box 3019, Morogoro, 67125 Tanzania

**Keywords:** Abscisic acid, AncA, Genome mining, *Inonotus sanghuang*, *Phellinus linteus*, Terpene synthase

## Abstract

**Background:**

The medicinal mushroom *Sanghuangporus* is renowned in East Asia for its potent therapeutic properties, attributed in part to its bioactive sesquiterpenoids. However, despite their recognized medicinal potential, the biosynthetic pathways and specific enzymes responsible for sesquiterpenoid production in *Sanghuangporus* remain unexplored, limiting opportunities to optimize their medicinal applications.

**Results:**

Sesquiterpenoids from four *Sanghuangporus* species were extracted through targeted isolation using mass spectrometry (MS)-based metabolomics, resulting in the discovery of six known abscisic acid-related compounds and one new compound, whose structure was determined through spectroscopic and computational analysis. We employed a natural product genome mining approach to identify a putative biosynthetic gene cluster (BGC) containing a sesquiterpene synthase gene, *ancA*, associated with the detected compounds. Biosynthetic pathways for these compounds were proposed based on an integrative approach combining BGC analysis and MS2 fragment-based dereplication. Further analyses revealed that the gene content and synteny of the *ancA* BGC are relatively well-conserved across *Sanghuangporus* species but less so outside the genus.

**Conclusions:**

A sesquiterpene synthase gene, its associated BGC, and the biosynthetic pathway for a group of detected abscisic acid-related sesquiterpenoids in *Sanghuangporus* were predicted through genomic and metabolic data analyses. This study addresses a critical gap in understanding the genetic basis of sesquiterpenoid production in *Sanghuangporus* and offers insights for future research on engineering metabolic pathways to enhance sesquiterpenoid production for medicinal use.

**Supplementary Information:**

The online version contains supplementary material available at 10.1186/s12864-025-11542-9.

## Background

*Sanghuangporus* (*Hymenochaetales*, *Basidiomycota*) is a genus of pharmaceutical mushrooms, esteemed in oriental medicine for its various properties. Before the systematic revision [1], *Sanghuangporus* species were referred to by various names. The type species *S. sanghuang*, commonly known as ‘sanghwang’ in Korea, ‘sanghuang’ in China, and ‘meshimakobu’ in Japan, has been called *Inonotus sanghuang*, *Phellinus igniarius*, and *Phellinus linteus* [2–5]. As such, chemicals derived from *Sanghuangporus* mushrooms were termed, for example, inoscavin, phelligrins, and phellilins, after their species names [6]. Currently, there are 20 accepted *Sanghuangporus* species, and of all, *S. baumii*, *S. sanghuang*, and *S. vaninii* are most commonly studied for their remedial effects. These species have reported antitumor, antioxidant, and anti-inflammatory properties [7–9], derived from secondary metabolites such as polysaccharides, polyphenols, and terpenoids. Among these diverse classes of compounds, sesquiterpenoids are frequently reported for *Sanghuangporus*, such as phelligridins, phellinene acids, and phellinulins [10–12].

Sesquiterpenoids are among the most commonly documented classes of terpenoids in basidiomycetes [13]. They participate in chemical defense against microbes and predators, such as insects [14–16]. In *Schizophyllum commune*, sesquiterpenoids are differentially expressed during the life cycle, suggesting their role in mating [17]. The biosynthesis of sesquiterpenoids, along with all other terpene derivatives, is generally initiated from farnesyl pyrophosphate (FPP; also known as farnesyl diphosphate, FDP). Transcripts and enzymes participating in the mevalonate (MVA) pathway that generates FPP in fungi have been well-defined and applied for *Sanghuangporus* [18, 19]. However, diverging downstream biochemical processes remain largely obscure despite the diversity of sesquiterpenoids produced. Only one sesquiterpene synthase (STS) gene, *SbTps1*, has been characterized in *S. baumii* [20], and its cyclization mechanism remains undetermined. Nonetheless, *Sanghuangporus* species produce various sesquiterpenoids, including cyclofarnesane derivatives resembling abscisic acid (ABA) [21, 22].

ABA and related compounds are produced by plants, fungi, and, to a limited extent, by bacteria. Nearly all higher plants synthesize ABA from FPP via the carotenoid pathway, which utilizes β-carotene produced through the methylerythritol phosphate pathway [23]. β-carotene is converted into zeaxanthin, which then undergoes a series of enzymatic reactions to form abscisic aldehyde and ultimately ABA. In bacteria, *Azospirillum brasilense* has been reported to produce ABA from malic acid [24], although the underlying biosynthetic mechanism remains unexplored. In fungi, ABA synthesis occurs via a direct pathway involving FPP cyclization to produce ionylideneethanol, followed by subsequent oxidation steps. Two variations of this pathway exist, depending on whether the species produce α-ionylideneethanol or γ-ionylideneethanol. The α-ionylideneethanol pathway is well-studied in *Botrytis cinerea*, where the *bcABA1* to *bcABA4* gene cluster governs ABA biosynthesis [25]. In contrast, the γ-ionylideneethanol pathway has primarily been investigated in *Cercospora* spp. [26, 27], though no specific genes or terpene cyclase enzymes have been identified for this route. 

Natural product genome mining has been used to explore the conservation and evolution of biosynthetic gene clusters (BGCs) in microorganisms [28]. This approach has revealed new classes of secondary metabolites, such as enediyne and isocyanide compounds, across various fungal lineages [29, 30]. In this study, natural product genome mining was employed to identify a potential STS gene and associated BGC in *Sanghuangporus.* Genomic and sesquiterpenoid metabolomic data from mycelial cultures of four species (*S. baumii*, *S. sanghuang*, *S. vaninii*, and *S. weigelae*) were analyzed. These species were selected for their availability, medicinal properties, and broad phylogenetic representation within the genus [31, 32]. Based on genomic and metabolomic data, we i) predicted the biosynthetic pathway for each sesquiterpenoid and the corresponding BGC, and ii) compared the BGC synteny among *Sanghuangporus* species and evaluated the conservation of the STS in basidiomycetes.

## Methods

### Study materials

Four *Sanghuangporus* species were collected between 2014 and 2022 in the Republic of Korea (Supplementary Material 3: Table S1). Cultural isolates were obtained from the fresh fruiting bodies using potato dextrose agar (PDA; Difco, Sparks, MD, USA) at 26℃. The identities of the fruiting bodies and the cultures were validated based on phylogenetic analysis (Supplementary Material 1: Supplementary Method 1; Supplementary Material 2: Fig. S1). Representative strains of four species were used in this study: *S. baumii* (KMRB15090420), *S. sanghuang* (KMRB14110725), *S. vaninii* (KMRB16060213), and *S. weigelae* (KMRB22010601). The specimen vouchers and culture strains are deposited as dried specimens and stocks (20% glycerol and distilled water), respectively, at the Seoul National University Fungus Collection (SFC).

### Chemical profiling using HR-ESI–MS

An analysis of extracts from four *Sanghuangporus* species was conducted using high-resolution electrospray ionization mass spectrometry (HR-ESI–MS). The HR-ESI–MS and UPLC-MS/MS experiments were carried out on an Orbitrap Exploris 120 mass spectrometer, which was connected to a Vanquish UHPLC system (ThermoFisher Scientific, Waltham, MA, USA). Chromatographic separation was achieved using a YMC Triart C18 column (100 × 2.1 mm, 1.9 μm) with a flow rate of 0.3 mL/min and maintained at 30 °C. The mobile phases consisted of water with 0.1% formic acid (A) and acetonitrile with 0.1% formic acid (B), and the elution was performed with a linear gradient ranging from 10 to 100% B over 10 min. Mass detection covered an *m/z* range of 200–2000, with the Orbitrap analyzer set to a resolution of 60,000 for full MS scans and 15,000 for data-dependent MSn scans. During mass spectrometry, the parameters included a spray voltage of 3.5/2.5 kV for positive ion modes, an ion transfer tube temperature of 320 °C, a vaporizer temperature of 275 °C for the HESI probe, and an RF lens setting of 70%. Ultrapure nitrogen (> 99.999%) served as the sheath and auxiliary gas for the HESI probe, set at 50 and 15 arbitrary units, respectively. The collision of ions in the Orbitrap detector was performed using a normalized higher-energy collision dissociation energy of 30%. MS/MS fragmentation data were obtained in data-dependent MSn mode, targeting the four most intense ions and employing a dynamic exclusion filter to prevent repeated ion fragmentation within 2.5 s of acquiring the MS2 spectrum*.*

### Feature-based molecular networking and Sirius analysis

The four raw data files were imported to MZmine 4.1.0 [33]. MS level (1) mass detection settings were performed by centroid setting the noise level at 8.0E5. Mass detection level (2) was set by centroid setting noise level of 1.0E1. Chromatogram builder settings included a minimum group size in the number of scans to be 4, minimum intensity for consecutive scans of 8.0E5, a minimum absolute height of 9.0E5, and scan-to-scan accuracy (*m/z*) at 1 ppm, 0.05 (*m/z*). Resolving was achieved using a retention time tolerance of 0.04 min, the minimum absolute height at 9.0E5, chromatographic threshold at 0.8. Minimum ratio of peak top/edge at 1.7, and Min number of data points of 4. 13 C isotope filtering parameters included *m/z* tolerance of 5.0 ppm, retention time tolerance of 0.03 min, and the most intense representative isotope to be considered as representative isotope. Isotopic peaks finder set parameters as chemical elements H, C, N, O, Cl, Br, P, and the *m/z* tolerance was 10.0 ppm, maximum charge of isotope *m/z* of 1. Aligning of feature list step set parameters as *m/z* tolerance at 5.0 ppm, retention time tolerance at 0.1 min, weight for RT at 2.0, and mobility weight at 1.0. Feature list filtering settings included minimum features in a row 1.0, minimum features in an isotope pattern 2, and *m/z* tolerance at 5.0 ppm. The parameters set in gap filling step included intensity tolerance at 0.2, *m/z* tolerance at 5.0 ppm, retention time tolerance at 0.1 min, and minimum data points at 2. Correlation grouping was performed by minimum feature height of 3.0E3, intensity threshold for correlation of 3.0E3, with feature correlation grouping. Ion identity networking setting included *m/z* tolerance of 10.0 ppm, min height of 1.0E3. The parameters set in MS/MS spectral networking included *m/z* tolerance at 5 ppm, minimum matched signals at 6, minimum cosine similarity 0.7. [34]. The feature list was exported to Sirius 6.0.0, where MS^2^ fragmentation analysis was performed to identify the molecular formula of the [M + H]^+^ adduct. Additionally, fingerprint prediction and structure database search were employed to enhance the accuracy of compound identification [35].

### Extraction, isolation, and structure determination of secondary metabolites

*Sanghuangporus* cultures were cultivated in PDA media (BD Difco, Sparks, MD, USA) and YEME solid media at room temperature. After four weeks, the strain cultures were extracted with ethyl acetate three times for chemical profiling. Among them, *S. weigelae* was selected for large-scale cultivation as the largest number of compounds were detected for this species. The culture was cultivated in YEME solid media at room temperature for the isolation of compounds. After 36 days, the cultures were extracted with ethyl acetate three times. *Sanghuangporus weigelae* extraction (2.5 g) was fractionated by medium pressure column chromatography (MPLC) over silica by a stepwise gradient of n-hexane-dichloromethane-MeOH (from 100:0:0, 50:50:0, 0:100:0, 0:99:1, 0:98:2, 0:97:3, 0:95:5, 0:90:10, 0:50:50 to 0:0:100) to obtain thirteen fractions (A ~ M fractions). Additional MPLC of isocratic method of CH_2_Cl_2_:MeOH (30:70) afforded compound 7 (803 mg). D fraction was subjected to reverse phase HPLC (Phenomenex, Luna Phenyl-Hexyl, 5 µ, 250 × 10.0 mm, isocratic aqueous 45% CH_3_CN, 2.0 mL/min, UV 210, 254, 280, and 365 nm) to obtain compound 6 (26.0 mg). E fraction obtained from the elution solvents 0:99:1 was subjected to reverse phase HPLC (Phenomenex, Luna C_18_, 5 µ, 250 × 10.0 mm, isocratic aqueous 45% CH_3_CN, 2.0 mL/min, UV 210, 254, 280, and 365 nm) to obtain compound 1 (1.6 mg), 2 (1.6 mg), 4 (0.7 mg). F fraction was subjected to reverse phase HPLC (Phenomenex, Luna C_18_, 5 µ, 250 × 10.0 mm, isocratic aqueous 50% CH_3_CN, 2.0 mL/min, UV 210, 254, 280, and 365 nm) to obtain compound 5 (23.1 mg). F fraction was subjected to reverse phase MPLC by a stepwise gradient of DW-MeOH (from 10:90 to 0:100) to obtain compound 3 (7.0 mg).

SHA- 1 (compound 1): white solid; [α]_d_^20^ = − 77.1 (c = 0.1, MeOH); UV (MeOH) λ_max_ (log ε) = 260 (4.10) nm; CD (MeOH) λmax (*Δ*ε) 253 (− 35.49), 315 (7.74) nm;; for ^1^H NMR (400 MHz, CDCl_3_)* δ*_H_ 6.08 (1H, d, *J* = 15.5 Hz, H- 8), 5.72 (1H, br s, H- 10), 5.67 (dd, *J* = 15.5 Hz, 10.5, H- 7), 2.92 (1H, dq, *J* = 6.9, 6.6 Hz, H- 5), 2.52 (1H, m, H_2_− 3a), 2.32 (1H, m, H- 6), 2.29 (1H, m, H_2_− 3b), 2.21 (3H, d, *J* = 1.1 Hz, H_3_− 12), 1.82 (1H, td, *J* = 14.0, 5.2 Hz, H_2_− 2a), 1.64 (1H, ddt, *J* = 14.0, 6.8, 2.2 Hz, H_2_− 2b), 1.34 (3H, s, H_3_− 15), 0.87 (3H, s, H_3_− 14), 0.83 (3H, d, *J* = 6.8 Hz, H_3_− 13); ^13^C NMR (100 MHz, CDCl_3_) *δ*_C_ 213.10 (C- 4), 171.65 (C- 11), 154.05 (C- 9), 137.58 (C- 8), 134.78 (C- 7), 118.05 (C- 10), 59.56 (C- 6), 43.66 (C- 5), 38.25 (C- 3), 36.02 (C- 2), 34.29 (C- 1), 29.75 (C- 14), 26.88 (C- 15), 14.28 (C- 12), 13.12 (C- 13); COSYs (CDCl_3_, H-# ↔ H-#) H- 2 ↔ H- 3, H- 5 ↔ H_3_− 13, H- 5 ↔ H- 6, H- 6 ↔ H- 7, H- 7 ↔ H- 8; HMBCs (CDCl_3_, H-# → C-#) H_a_− 2 → C- 1 and C- 15; H_b_− 3 → C- 2 and C- 4; H- 5 → C- 4, C- 6, C- 7 and C- 13; H- 6 → C- 1, C- 5 and C- 7; H- 7 → C- 5, C- 6 and C- 9; H- 8 → C- 6, C- 9, C- 10 and C- 14; H- 10 → C- 8 and C- 14; H_3_− 12 → C- 8, C- 9, C- 10 and C- 11; H_3_− 13 → C- 4, C- 5 and C- 6; H_3_− 14 → C- 1, C- 2, C- 6 and C- 15; H_3_− 15 → C- 1, C- 2, C- 6 and C- 15; (+)-HR-ESI–MS *m/z* 251.1648 [M + H]^+^, calc for C_15_H_23_O_3_, 251.1642.

### High molecular weight genomic DNA extraction

*Sanghuangporus* cultures were grown in 150 ml static or shaking (150 min^−1^) potato dextrose broth (PDB; Difco, Sparks, MD, USA) at 26℃ for 5–10 days for the genomic DNA extraction. The grown mycelia were dehydrated using a GAST vacuum pump (Michigan, USA), and high-molecular-weight DNA was extracted using the HMW DNA extraction kit (Wizard, Promega, WI, USA) or through a modified cetyltrimethylammonium bromide (CTAB) protocol (Supplementary Material 1: Supplementary Method 2). The extracted genomic DNA was quantified using Nanodrop 2000 spectrophotometer (Thermo Fisher Scientific, Waltham, MA, USA) and Quantus Fluorometer (Promega, Madison, WI, USA).

### Library preparation and DNA sequencing

Library preparation and sequencing were conducted at the National Instrumentation Center for Environmental Management (NICEM), Seoul National University (Seoul, Rep. of Korea) for Oxford Nanopore Technologies (ONT), CJ Bioscience (Seoul, Rep. of Korea) for PacBio sequencing, and Macrogen (Seoul, Rep. of Korea) for Illumina sequencing.

ONT libraries were constructed using the ONT ligation sequencing kit and Circulomics Short Read Eliminator (SRE) Kit. ONT sequencing was performed on PromethION P24 using a R9.4.1 cell and MinKnoW 21.11.7 software for *S. baumii* and *S. sanghuang*, and R10.4.1 cell for *S. vaninii*. Genomic data for *S. weigelae* was achieved through library preparation (5 kb) and PacBio sequencing (Sequel II system). Different sequencing platforms were used based on resource availability. Illumina NovaSeq 6000 platform was used for polishing the four long-read genomic data. Paired-end sequencing was performed using 2 × 150 bp libraries.

### Genome assembly and annotation

Raw nanopore reads from R9.4.1 cell were basecalled by Guppy 6.1.3 (https://community.nanoporetech.com), and reads from R10.4.1 cell were basecalled by Dorado 0.4.1. (https://github.com/nanoporetech/dorado), both with super accurate model. Hifi reads were generated by PacBio CCS (https://github.com/PacificBiosciences/ccs) for PacBio sequencing. Adapters were trimmed by porechop 0.2.4 (https://github.com/rrwick/Porechop) for nanopore reads, HiFiAdapterFilt 2.0.1 [36] for PacBio reads, and fastp 0.23.2 [37] for Illumina reads (Supplementary Material 3: Table S2).

Nuclear genome assembly was performed with NextDenovo 2.5.0 [38] for nanopore sequence data, PECAT 0.0.3 [39] when high heterogeneity was found, and hifiasm 0.19.5 [40] for PacBio data. Polishing was performed with four rounds of Racon 1.5.0 [41] for long reads mapped by minimap2 2.24 [42], one round of medaka 1.6.0 (https://github.com/nanoporetech/medaka) (only for nanopore sequencing), and four rounds of Hapo-G 1.3.1 [43] for Illumina reads. Jellyfish k-mer 2.3.0 [44] and GenomeScope 2.0 [45] were performed to construct a k-mer histogram and evaluate diploidy of the genomes. When diploids were observed in the k-mer plot, haplotypes were merged with Purge Haplotigs 1.1.2 [46]. The quality of the genomes was assessed with Tapestry 1.0.0 [47] using telomere “CTGGTG”, and UFCG 1.0.5 [48]. Low-depth contigs or contigs with low GC contents were regarded as non-nuclear genomic data and filtered.

RepeatModeler 2.0.4 [49] and RepeatMasker 4.1.2 [50] were performed to mask repeats of the genomes before annotation. Structural annotation was conducted with BRAKER3 3.0.3 [51] with --fungus option. For non-coding RNA sequences, tRNAs were found using tRNAscan-SE 2.0.9 [52], and rRNAs were annotated using Infernal 1.1.4 [53] with Rfam 14.8 [54] database. For functional annotations, we employed EggNOG-mapper 2.1.8 [55] using EggNOG 5.0 database [56] and MMseqs2 13.45111 [57] option, searching for e-value of 1E-5 or lower. Then we used Funannotate 1.8.15 (https://github.com/nextgenusfs/funannotate) through “iprscan” (InterProScan5 [58] wrapper) and UPIMAPI 1.12.2 [59]. All results were then merged with an “annotate” function in Funannotate. The final genome sequences have been deposited in NCBI under the accessions JBKPUN000000000 (*S. baumii*), JBKPUO000000000 (*S. sanghuang*), JBKPUM000000000 (*S. vaninii*), and JBKPUL000000000 (*S. weigelae*).

### Biosynthetic gene cluster and pathway prediction

A BLASTp 2.15.0 [60] search was conducted to find orthologs of the core STS gene, *ancA*, from *Antrodia cinnamomea* [61] in the *S. weigelae* genome. Additionally, BGCs producing secondary metabolites were predicted in the *S. weigelae* genome using the full-featured antiSMASH 7.0.0 [62] with --taxon fungi option. An additional database-independent BGC search was performed with DeepBGC 0.1.31 [63] to find putative BGCs. Of all the BGCs identified, two included the core STS gene. One BGC was selected based on its DeepBGC score, predicted biological activity, and the types of secondary metabolites it was predicted to produce. The genes in the selected BGC (hereafter referred to as the *ancA* BGC) were transcribed in silico into protein sequences and folded using the Alphafold 3 server [64]. For the AncA protein, an additional structure incorporating an Mg^2+^ ion was also predicted. A structural similarity search was performed for all proteins using Foldseek 7.04e0ec8 [65], against Alphafold Swiss Prot, PDB [66], and UniProt 50 [67] databases.

Based on our metabolomic profiles of *Sanghuangporus*, a putative biosynthetic pathway was constructed. Some steps were verified through reference searches, while the others were predicted based on possible biochemical reactions. From the *ancA* BGC, each predicted protein was tested in silico for its binding affinity against all metabolites in the putative pathway using Vina-GPU 2.1 [68]. All SMILES formulas of metabolites in the pathway were extracted using ChemSketch (https://www.acdlabs.com/resources/free-chemistry-software-apps/chemsketch-freeware/) and converted into 3-dimensional structures using RDKit (https://www.rdkit.org/) and Open Babel [69]. Molecular docking was performed using 64 spatial sections to cover the entire protein. The section resulting in the lowest binding energy was identified. The terpene cyclase domain of the AncA protein (the primary STS) and FPP was visualized using PyMOL software (version 3.1.1, Schrödinger, LLC, Portland, U.S.A.) 3.1.1 [70]. Similarly, the phosphatase domain of Mg^2+^-postulated AncA and the diphosphate-attached compound **9** (intermediate) were visualized using the same method. A heatmap depicting the lowest binding energy for all tested proteins was generated using the python *seaborn* library.

### Conservation analysis of AncA and the BGC

The top 100 hits of orthologous sequences of the AncA amino acid sequence were collected from the NCBI BLASTp results using *Sanghuangporus* AncA sequences as the query. After a preliminary round of phylogenetic analysis (Supplementary Material 2: Fig. S2), only the orthologous protein sequences were selected for the final analysis. For the reference NCBI *Sanghuangporus* genomic data, AncA sequences that were not available in the BLASTp database were manually included for the final analysis. All reference and query sequences were aligned using MAFFT version 7 [71] in Geneious Prime 2024.0.5 (https://www.geneious.com) with the BLOSUM62 substitution matrix and a gap open penalty of 3. After a model test of the alignment using ModelTest-NG [72], the JTT-DCMUT + I + G4 model was selected for the phylogenetic analysis. A maximum likelihood tree was inferred using RAxML v. 8.2.12 [73] with 1,000 bootstrap replicates.

Species phylogenomic trees were inferred to analyze the phylogenetic relationship among studied strains. Fungal core genes indicated in the UFCG database were extracted from genomes and aligned using the UFCG pipeline [48]. Core gene alignments were concatenated with SuperCRUNCH 1.3.2 [74], and the phylogenomic tree was built using IQTREE2 [75] with 1,000 ultrafast bootstraps [76] and the ModelFinder [77] option. For the synteny analysis of the *ancA* BGC across *Hymenochaetaceae*, reference genomic data assembled into fewer scaffolds were selectively collected from the NCBI database. From these reference data, the *ancA* BGC was located using cblaster 1.3.19 [78]. Clinker [79] was used to visualize the synteny of each strain.

## Results

### Isolation and structural elucidation

**Fig. 1:**
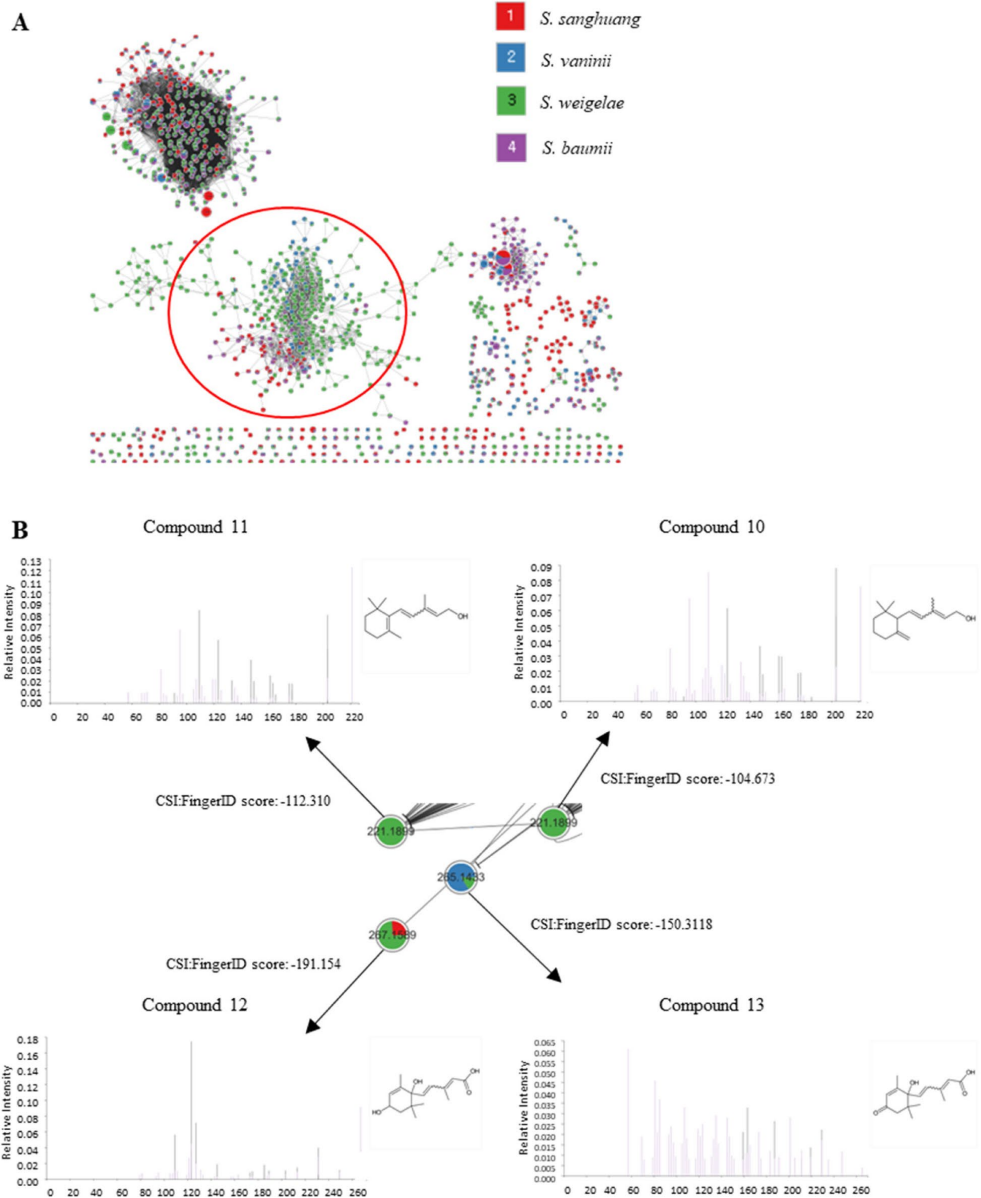
**A** Full molecular network highlighting the target ABA-related compound cluster. **B** MS2 fragmentation of the nodes within the target cluster was analyzed, with black arrows indicating matched fragments. This analysis was based on molecular formula annotation (M + H).^+^ and substructure annotation using Sirius 6.0.0 (11–13)

**Fig. 2:**
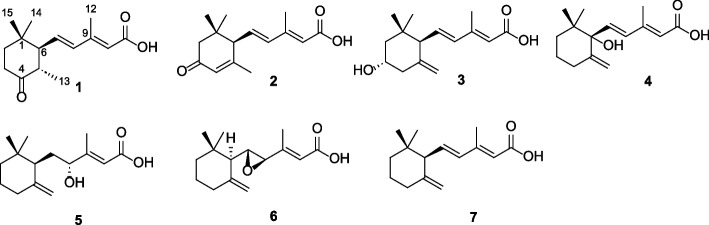
Sesquiterpenoids isolated from *Sanghuangporus* species

Feature-based molecular networking (Fig. 1A), combined with Sirius MS2 fragmentation base compound class annotation (Fig. 1B), effectively identified the target cluster of sesquiterpenoids. Targeted isolation of *Sanghuangporus* spp. extracts afforded seven compounds, and their structures were determined by comparing their spectroscopic data (Fig. 2) with those in the references [80–82].

The new sesquiterpenoid (1), named as SHA- 1, had a molecular formula of C_15_H_22_O_3_, based on the (+)HRESIMS, with 5 degrees of unsaturation. Its ^1^H NMR spectrum (Table 1) revealed three aliphatic protons [*δ*_H_ 6.08 (d, *J* = 15.5 Hz), 5.72 (br s), 5.67 (dd, *J* = 15.5, 10.5 Hz)]. The ^13^C NMR spectrum indicated the presence of one carboxylic acid (*δ*_C_ 171.6), one ketone (*δ*_C_ 213.0), and two double bonds (*δ*_C_ 154.0, 137.6, 134.8, and 118.0). Therefore, the presence of one ring in the structure was evident to account for the unsaturation degree. Interpretation of ^1^H-^1^H COSY spectra afforded two substructures, one from H_2_− 2 (*δ*_H_ 1.82 and 1.64) to H_2_− 3 (*δ*_H_ 2.52 and 2.29) and the other from H_3_− 13 (*δ*_H_ 0.83) to H- 8 (*δ*_H_ 6.08) through H- 5 (*δ*_H_ 2.92), H- 6 (*δ*_H_ 2.32), and H- 7 (*δ*_H_ 5.67), consecutively. The connection of two substructures and positions of the functional groups were confirmed by analysis of HMBC spectrum. The strong HMBC correlations of H_3_− 12 (*δ*_H_ 2.20) with C- 8 (*δ*_C_ 137.6), C- 9 (*δ*_C_ 154.0), C- 10 (*δ*_C_ 118.0), and C- 11 (*δ*_C_ 171.6) indicated the presence of a methyl-diene chain which is further connected with the carboxylic acid. Further correlations of two tertiary methyls (*δ*_H_ 1.34 and 0.87) with C- 2 (*δ*_C_ 34.3) and C- 6 (*δ*_C_ 59.6), along with those of H_3_− 13 (*δ*_H_ 0.83) with the ketone carbon and C- 6, confirmed that it had a 4-dimethyl- 1-methyl cyclohexanone with the 3-methyl-pentaenoyl attachment. The geometry of the two double bonds in the aliphatic chain was determined to be both *trans*, based on their coupling constants (*J* = 15.5 Hz) and NOESY correlations: H- 7 with H_3_− 12 and H- 8 and H- 10. The absolute stereochemistry of C- 5 and C- 6 was determined by a quantum-mechanic based molecular calculation method such as ECD calculation, DP4 calculations after establishment of relative one by NOESY analysis.

**Table 1:** ^1^H and ^13^C NMR data of compound 1

No	*δ*_C_, Carbon type^a^	*δ*_H_ (multi., *J* in Hz)^a^
1	34.29, C	-
2	36.02, CH_2_	1.82 (td, 14, 5.2), 1.64 (ddt, 14, 6.8, 5.2)
3	38.25, CH_2_	2.52 (m), 2.29 (m)
4	213.1, C	-
5	43.66, CH	2.92 (dq, 6.9, 6.6)
6	59.56, CH	2.32 (m)
7	134.78, CH	5.67 (dd, 15.5, 10.5)
8	137.58, CH	6.08 (d, 15.5)
9	154.05, C	-
10	118.05, CH	5.72 (br s)
11	171.65, C	-
12	14.28, CH_3_	2.21 (d, 1.1)
13	13.12, CH_3_	0.83 (d, 6.8)
14	29.75, CH_3_	0.87 (s)
15	26.88, CH_3_	1.34 (s)

^a^Data measured at 400 and 100 MHz in CDCl_3_

**Fig. 3:**
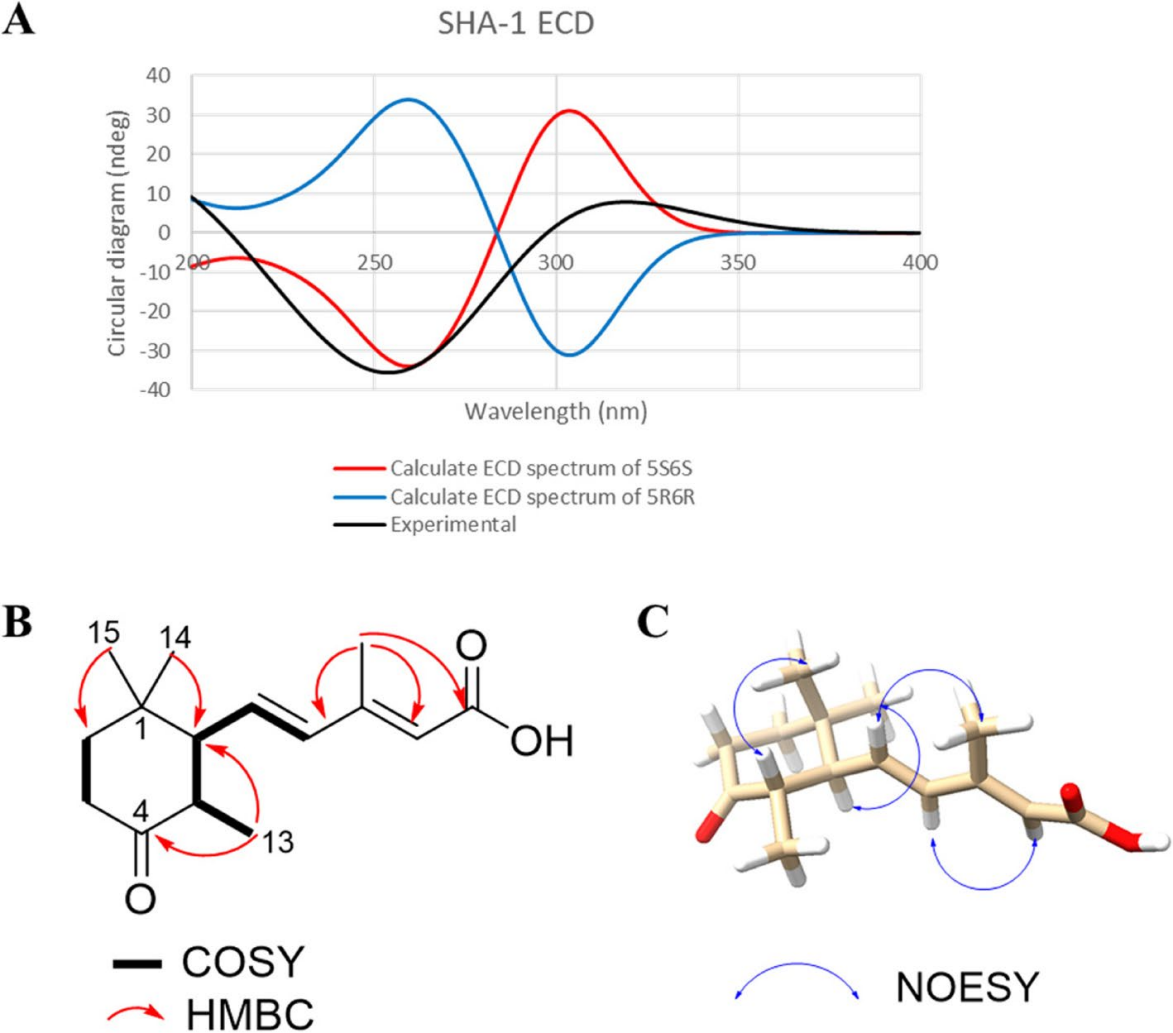
**A** Calculated ECD data for compound 1 (SHA- 1). **B** Key ^1^H-^1^H COSY (bold lines) and HMBC (red arrows) correlations. **C** Key NOESY correlations (indicated by blue arrows)

In the NOESY interpretation, it was noted that the axial or pseudo-axial positions of H_3_− 15 (*δ*_H_ 1.34) and H_3_− 14 (*δ*_H_ 0.87) were assigned based on the comparison of ^1^H NMR data with that of a structurally similar reference compound [83]. The analysis of NOESY correlations established the spatial proximity between H- 5 and H_3_− 15, as well as H- 6 and H_3_− 14, while no correlation between H- 6 and H_3_− 15 was observed. This indicates that the methyl group at C- 5 and the 3-methyl-pentaenoyl group at C- 6 are on the opposite face of the molecule. However, the *J*-value of 6.9 Hz between H- 5 and H- 6 is a bit small for this. To resolve the ambiguity, DP4 probability analysis was performed on four possible stereoisomers (5R*,6R*; 5R*,6S*; 5S*,6R*; 5S*,6S*). The analysis revealed that the isomer with the 5S*,6S* configuration had a 100.0% probability, confirming the relative configuration established by NOESY. To further establish the absolute configuration, its experimental ECD spectrum was compared with those of calculated ECD for the two possible isomers (5*R*,6*R* and 5*S*,6*S*), confirming the final configuration. As a result, negative Cotton effects at 253 nm together with positive Cotton effects 315 nm in the experimental ECD spectrum agreed with that of the isomer with 5*S*,6*S* (Fig. 3A). Thus, the structure of the new compound (**1**) including stereochemistry was completely established (Fig. 3B; Supplementary Material 1: Supplementary Methods 3–4).

### Genome assembly

High-quality genomic data were obtained for four *Sanghuangporus* species. The genome size ranged from 31.9 to 36.2 Mb, and the GC content ranged from 47.80% to 48.11%. The genomes showed BUSCO completeness ranging from 91.0% to 93.7%, and all 55 nuclear UFCG genes were identified. Indices indicating genome contiguity were as follows: N50, 2.52–3.02 Mb; 17–25 contigs; and telomeres present in 23.5% to 73.5% of the genome. Diploidy was also well solved, and read depth, calculated from tapestry, corresponded to the contigs. Across the genomes, 9,664 to 10,126 protein-coding genes were annotated.

### Predicted BGCs of *Sanghuangporus*

The genomic data of *S. weigelae* were selected as a representative for all subsequent analyses. In the core STS analysis, the sequence of ACEPAI_7410 and ACEPAI_4311 exhibited 54.1% and 43.2% protein identity, respectively, to the AncA of *Antrodia cinnamomea* (= *Taiwanofungus camphoratus*) V7 (GCA_022598595), which was the first species to report AncA as an STS. Other hits showed very short alignments (less than 100 bases) and were, therefore, considered false-positive matches.

An antiSMASH 7.0.0 search of the four *Sanghuangporus* genomes resulted in 19 BGCs for *S. sanghuang*, 19 for *S. baumii*, 20 for *S. weigelae*, and 25 for *S. vaninii*. Gene ACEPAI_4311 was included in one of the BGCs predicted by antiSMASH 7.0.0, but gene ACEPAI_7410 was not found. On the other hand, a DeepBGC search of the *S. weigelae* genomic data predicted 373 BGCs. Among them, two gene clusters included ACEPAI_4311 and ACEPAI_7410, respectively (Table 2). The putative BGC containing ACEPAI_7410 had a DeepBGC score of 0.94022 with 0.44 terpene prediction, while the putative BGC containing ACEPAI_4311 had a DeepBGC score of 0.58855 with a 0.7 polyketide prediction. Thus, we concluded that ACEPAI_7410 and the putative BGC containing it are the actual putative BGC producing compounds found in the HPLC analysis. The putative BGC was 112 kbp in length, and contained 172 genes, included a STS gene, *ancA*. The predicted product type for the *ancA* BGC was highest for terpene (0.44), compared to alkaloid (0.2), NRP (0.02), polypeptide (0.1), RiPP (0.07), saccharide (0.07), and others (0.12). The predicted product activity of the *ancA* BGC was highest for antibacterial (0.61), compared to cytotoxic (0.1), inhibitor (0.34), and antifungal (0.07). The initial 172 genes were reduced to 41 genes after wrapping the annotation result from BRAKER3.

**Table 2:** DeepBGC results for predicted AncA-based sesquiterpenoid synthesis in *Sanghuangporus*

Gene		**ACEPAI_7410**	ACEPAI_4311
Location		contig6 63,208–175,401	contig2 2,757,758–2,805,477
DeepBGC score		**0.94022**	0.58855
Predicted biological activity	Antibacterial	**0.61**	0.38
	Cytotoxic	0.1	0.03
	Inhibitor	0.34	0.26
	Antifungal	0.07	0.26
	Alkaloid	0.2	0.01
	NRP	0.02	0
	Other	0.12	0.14
Predicted secondary metabolite type	Polyketide	0.1	**0.7**
	RiPP	0.07	0.1
	Saccharide	0.07	0.02
	Terpene	**0.44**	0.04

### Functions of genes in ancA BGC

**Fig. 4:**
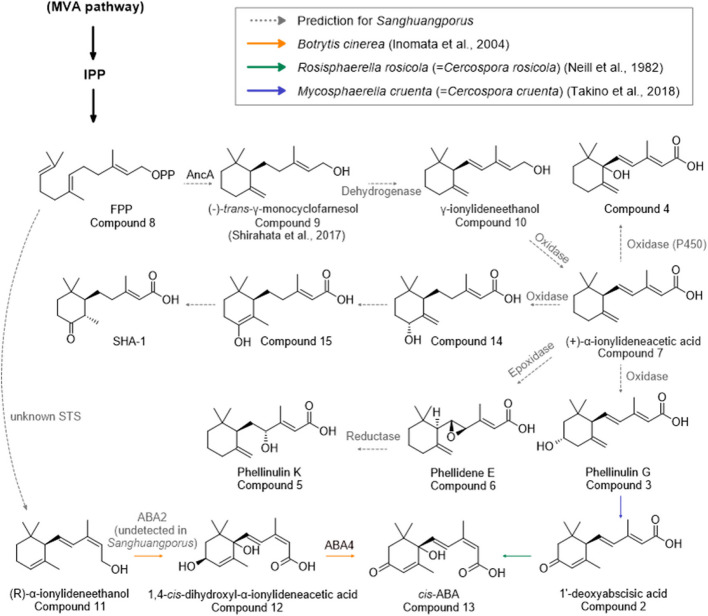
Putative biosynthetic pathway of ABA-related sesquiterpenoids in *Sanghuangporus*. Metabolomic data is from *S. weigelae* (KMRB22010601). Intermediate compounds 9, 14, and 15 were not detected

**Fig. 5:**
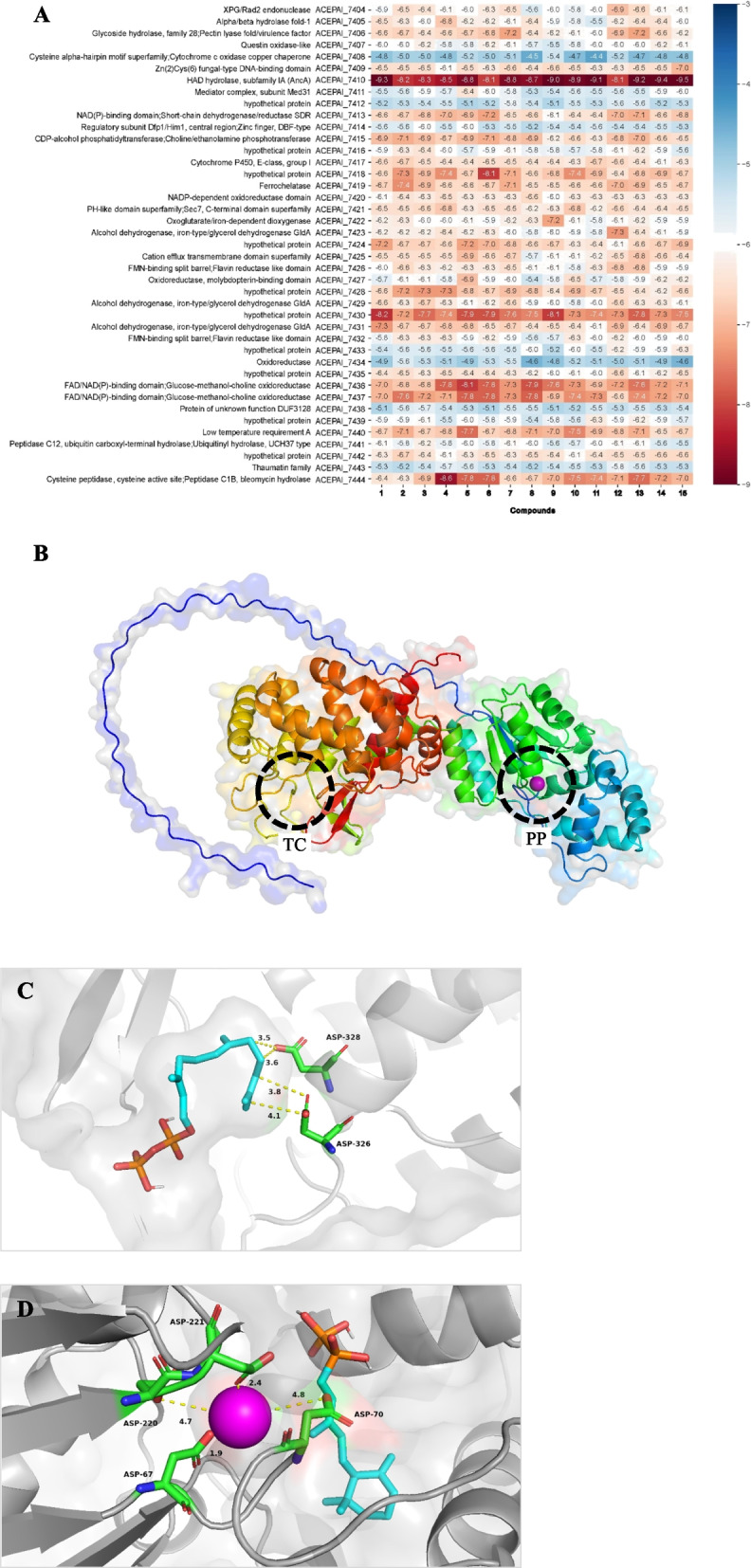
Protein–ligand binding of enzymes derived from *ancA* BGC and detected metabolites in *Sanghuangporus*.** A** Heatmap for the best (minimum) binding energy values (ΔG, kcal/mol) for protein-metabolite pairs. Protein functions were predicted based on the Swiss Prot database. **B** Predicted structure of AncA. The terpene cyclase and pyrophosphatase domains are indicated as ‘TC’ and ‘PP’, respectively. Chelating metal ion Mg^2+^ is shown in pink for the pyrophosphatase domain. **C** Predicted FPP binding at the terpene cyclase domain of AncA. **D** Predicted FPP binding at the pyrophosphatase domain of AncA. Chelating metal ion Mg^2+^ is shown in pink

Among the 41 genes included in the putative *ancA* BGC, some were further predicted to participate in the synthesis of ABA-related sesquiterpenoids detected from the metabolomic profiles of *Sanghuangporus* (Fig. 4). The functions of the genes in the *ancA* BGC were assessed through sequence similarity searches (Fig. 5A). The similarity search annotated meaningful functions for 31 of the 41 genes, while the remaining 10 genes (ACEPAI_7412, ACEPAI_7416, ACEPAI_7418, ACEPAI_7424, ACEPAI_7428, ACEPAI_7430, ACEPAI_7433, ACEPAI_7435, ACEPAI_7439, and ACEPAI_7442) were predicted to encode hypothetical proteins. Further analysis using Foldseek predicted the functions of all 10 proteins for the corresponding genes (Supplementary Material 3: Tables S3–S5). Additionally, another possible STS was identified using Foldseek; the protein of ACEPAI_7418 showed 19.3% structure identity and 3.223E-9 e-value with terpene synthase DEP1 (Supplementary Material 3: Table S3).

### Sesquiterpenoid biosynthesis pathway prediction with metabolomic analyses

The biosynthesis pathway of the isolated compounds was proposed based on the genomic data and the chemistry of the isolated compounds (Fig. 4). Although not all compounds presented in the proposed pathway were isolated in this investigation, metabolites 10–13 were identified to be present in the extracts and assigned using the feature-based molecular networking data with a minimum cosine similarity of 0.7 and Sirius 6.0.0 compound annotation based on MS2 fragment analysis (Fig. 1).

Compound 8 (FPP), generated by the MVA pathway, is converted by the enzyme AncA into compound 9. This intermediate is then dehydrogenated and subsequently oxidized to yield compound 7, which undergoes further modifications catalyzed by oxidase (P450) and epoxidase, leading to the formation of compounds 3, 4, and 6. Compound 6 (phellidene E) is further reduced by a reductase to produce compound 5 (phellinulin K). Oxidation of the cyclohexane ring in compound 3 generates the β-methyl-α, β-unsaturated carbonyl group in compound 2 (1'-deoxyabscisic acid). Subsequent *cis*–*trans* isomerization leads to the generation of compound 13 (*cis*-ABA). Additionally, compound 11, identified through metabolic analysis, is predicted to be produced from compound 8 (FPP) by an unknown terpene cyclase. Compounds 12 and 13 are proposed to be produced by an unknown enzyme and ABA4, respectively. Ultimately, compound 1 (SHA- 1) is anticipated to be produced via enol-keto modification and the action of an oxidase from compound 7 ((+)-γ-ionylideneacetic acid) through compounds 14 and 15.

### Comparative synteny analyses

**Fig. 6:**
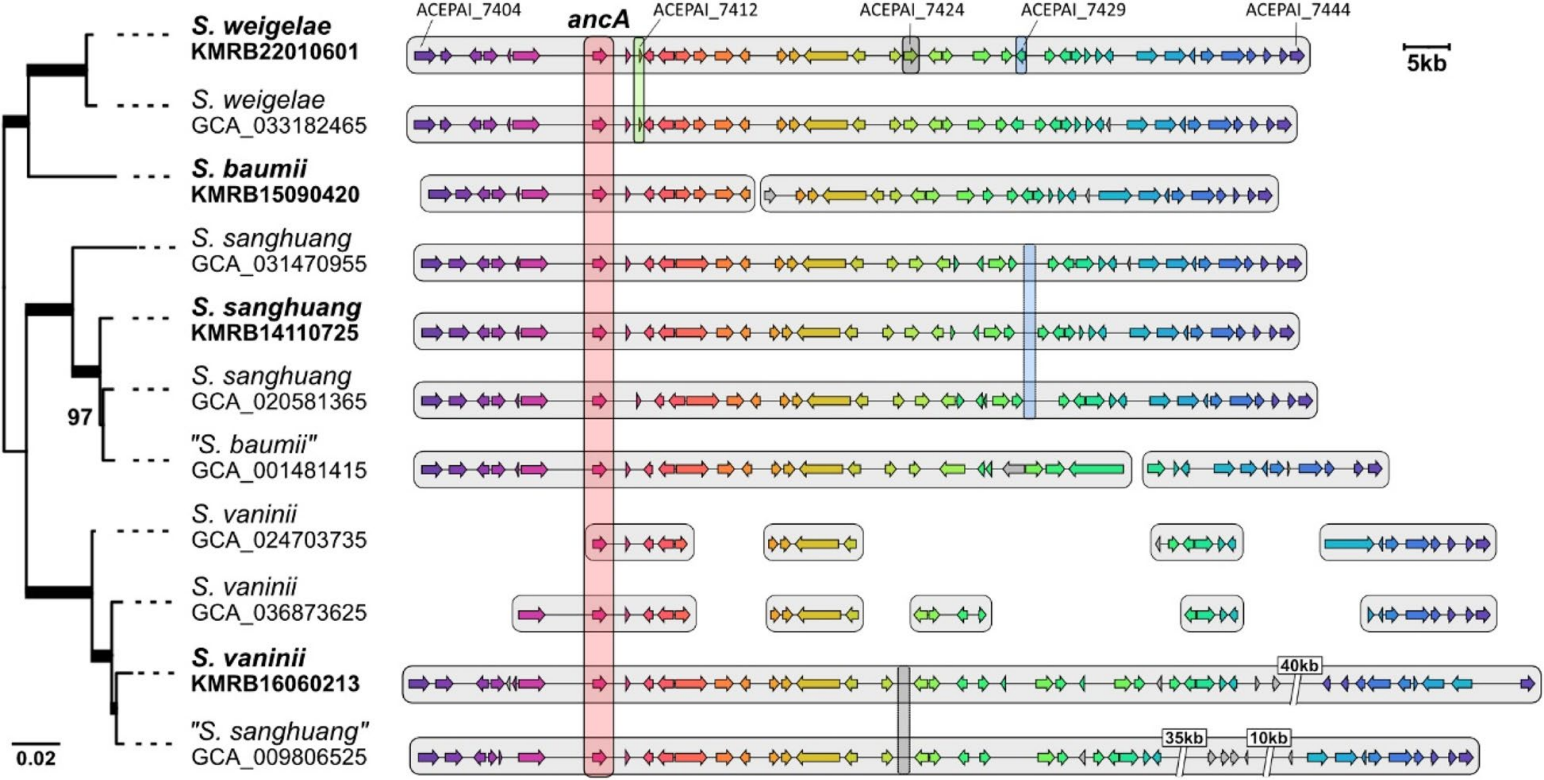
Synteny analysis of *ancA* BGC across *Sanghuangporus* species. The species tree on the left has been inferred based on 59 single copy genes. Strains newly analyzed in this study are bolded. Strains of possibly misidentified species names are annotated with quotation marks (“”). Genes are enclosed by gray boxes based on contigs/scaffolds. Comparative examples of exclusively present/absent or repositioned genes are labelled on top and indicated by colored shades. Predicted functions of encoding proteins for each gene are available in Fig. 5A

The gene content and synteny of the *ancA* BGC are relatively well-conserved across *Sanghuangporus* species (Fig. 6). Two *S. vaninii* genomes (GCA_024703735 and GCA_036873625) exhibited incomplete synteny, possibly due to fragmentation of the assembled genomes. *Sanghuangporus baumii* KMRB15090420 possesses the BGC across two contigs (2 and 8). Notable differences exist among species. For example, ACEPAI_7412 is exclusively found in *S. weigelae*; ACEPAI_7424 is missing in *S. vaninii* (GCA_009806525 is annotated as *S. sanghuang* but is phylogenetically identified as *S. vaninii*); and ACEPAI_7429 is repositioned in *S. sanghuang*. Overall, the conservation of synteny mirrors the species’ phylogenetic relationships.

The BGC becomes more disorganized outside the genus as the species becomes phylogenetically more distant from *Sanghuangporus* (Supplementary Material 2: Fig. S3). For the most distant species, *Pyrrhoderma noxium* (GCA_002287475), more than half (24) of the genes were absent. Nevertheless, the core *ancA* gene (ACEPAI_7410) and nine other genes (ACEPAI_7414, ACEPAI_7419, ACEPAI_7420, ACEPAI_7421, ACEPAI_7434, ACEPAI_7435, ACEPAI_7438, ACEPAI_7439, and ACEPAI_7441) were conserved across *Hymenochaetaceae*.

### Conservation of AncA in basidiomycetes

**Fig. 7:**
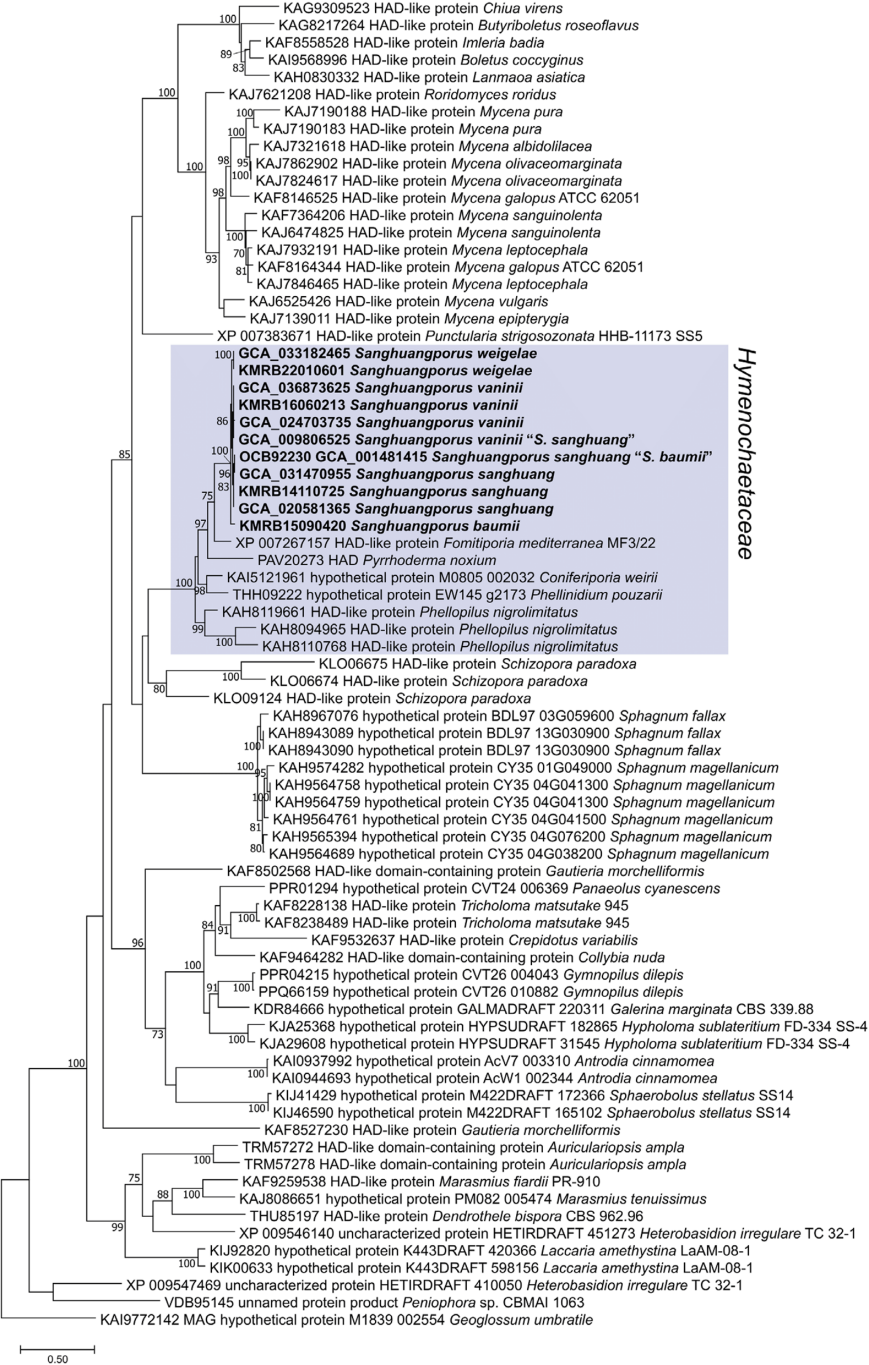
Maximum likelihood phylogenetic tree of *Sanghuangporus* AncA orthologous protein sequences. Species names in quotation marks (“”) are names in GenBank. Sequence of *Geoglossum umbratile* (*Ascomycota*) has been selected as an outgroup. Node bootstrap support values ≥ 70 are indicated

Of the 34 available *Hymenochaetales* genomes on NCBI (accessed: 2024–05–28), 11 haloacid dehydrogenase (HAD)-like proteins were detected through NCBI BLASTp hits of *Sanghuangporus* AncA amino acid sequences, including one from *S. baumii* strain 821 (GCA_001481415). Protein sequences from *Hymenochaetaceae*, which includes the entire *Hymenochaetales* except *Schizopora paradoxa* (reidentified as *Xylodon ovisporus* [84]), were monophyletically grouped with 100 bootstrap support (Fig. 7). Multiple copies of highly similar proteins from peat moss (*Sphagnum* spp.) were detected and included in the phylogenetic analysis, forming formed a clade outside the *Hymenochaetales* clade. Furthermore, groups of paralogous AncA proteins were also detected from diverse basidiomycetes lineages (Supplementary Material 2: Fig. S2).

### Protein-metabolite binding prediction

Binding affinity analysis was conducted using Vina-GPU 2.1 to predict the likelihood of protein-metabolite interactions. The best (minimum) binding energy value (ΔG, kcal/mol) ranged from − 4.4 and − 9.5 kcal/mol (Fig. 5A). The protein products of ACEPAI_7410 (AncA), ACEPAI_7430, ACEPAI_7436, ACEPAI_7437, ACEPAI_7440, and ACEPAI_7444 exhibited relatively high binding affinities across all analyzed secondary metabolites. Notable pairings included ACEPAI_7418 (a hypothetical protein) with compound **6**, and ACEPAI_7444 (a peptidase) with compound 4. The protein structure of ACEPAI_7410, predicted using Alphafold 3, showed a high structural similarity to AncA (Fig. 5B), with a PyMOL alignment score of 1242.500 and an RMSD of 2.649. In the terpene cyclase domain of AncA, two aspartate residues were found to interact with FPP (Fig. 5C). In the phosphatase domain, Mg^2+^-postulated AncA demonstrated an interaction with diphosphate-attached compound **9**, showing a binding energy of − 7.5 kcal/mol (Fig. 5D).

## Discussion

Feature-based molecular networking efficiently identified a large cluster of nodes predicted to be ABA-related sesquiterpenoides. Through chromatographic methods focused on isolating this cluster, we successfully obtained six known compounds (2–7) and identified one novel compound, SHA- 1 (compound 1). SHA- 1 is distinguished by a ketone functional group and a methyl group. The methyl group appears as a doublet in its NMR spectrum, unlike the other isolated compounds, which feature either a singlet methyl group or an exomethylene group at the corresponding position. This metabolomic information was effectively used to propose an ABA-related biosynthetic pathway, combining genome sequencing data, reference, and analysis of molecular formulas with MS2 fragmentation similarity matching using Sirius 6.0.0.

The sesquiterpenoid biosynthesis pathway was predicted based on the genomic data and the chemistry of the isolated compounds. This prediction was supported by previous studies. The core STS enzyme AncA converts compound 8 (FPP) to compound 9, which has been previously isolated from *Phellinus linteus* [81]. The transition from compound 3 to compound 2 has been reported for *Rosisphaerella cruenta* (= *Cercospora cruenta*) [85]. *Cis*–*trans* isomerization between compounds 2 and 13 has been observed in *R. rosicola* (= *C. rosicola*) [86]. While the exact mechanism and STS behind the conversion of FPP to compound 11 (α-ionylideneethanol) is unrevealed, the enzymes responsible for the syntheses of compounds 12 and 13 are known in *Botrytis cinerea*, which are ABA2 and ABA4, respectively [25, 87]. In *Botrytis cinerea*, four biosynthetic genes, *bcABA1* to *bcABA4*, exist in a BGC [88]. An orthologous *ABA4* gene has been detected in *Sanghuangporus* [89]. The *ABA4* gene encodes a protein (short-chain dehydrogenase/reductase) that synthesizes ABA from 1,4-*trans*-dihydroxyl-α-ionylideneacetic acid. However, no gene homologous to that of ABA2 was identified for our studied strains. Additional studies are required to identify the enzyme responsible for the conversion of compound 11 to compound 12.

No related genes were previously known for the downstream γ-ionylideneethanol (compound 10) pathway in the Kingdom *Fungi*, even for *Cercospora*, which has been exceptionally studied for the ABA synthesis pathway based on γ-ionylideneethanol [26, 90, 91]. The deep learning-based BGC search, deepBGC, allowed us to detect a putative BGC for the ABA-related compound syntheses using γ-ionylideneethanol based on in silico methods. In addition, protein-folding and foldseek allowed us to predict the protein functions through structural comparison using a well-established database from fungal species that may be evolutionarily distant from *Sanghuangporus* (e.g. ACEPAI_7418 in Supplementary Material 3: Table S3). Furthermore, molecular docking calculation allowed us to predict which genes in the *ancA* BGC are likely to be involved in the biosynthetic pathway of the detected ABA-related sesquiterpenoids, such as ACEPAI_7436 and ACEPAI_7437 (Fig. 5A). Other genes with low binding affinity are assumed to be involved in genetic expression or signaling, according to their functions. For example, ACEPAI_7411, encoding for a putative mediator complex, may be involved in regulating RNA polymerase II-dependent transcription. The ACEPAI_7421 gene encodes for an enzyme comparable to GRP1. This enzyme has a pleckstrin homology (PH) domain that is highly similar to yeast Sec7 protein, which catalyzes guanine nucleotide exchange of ADP ribosylation exchange factor, ARF [92, 93]. Given that ARF proteins regulate membrane trafficking, the GRP1-like protein may participate in activating signaling pathways.

The core protein of *ancA* BGC, the terpene cyclase AncA, is a HAD-like protein, a primary enzyme that converts FPP to (*R*)-*trans*-γ-monocyclofarnesol in *A. cinnamomea* (= *T. camphoratus*). It contains two domains, terpene cyclase and pyrophosphatase, which catalyze the cyclization of FPP and remove pyrophosphate, respectively [61]. The *ancA* gene is part of a BGC with genes *ancB*, *ancC*, *ancD*, and *ancE* that are responsible for the production of antrocin and its relatives [61]. These compounds are responsible for diverse anti-cancer effects [94–96]. Similarly, *ancA* and neighboring genes in the BGC are deemed to be involved in the synthesis of various pharmaceutical compounds in *Sanghuangporus*. Antibacterial, anti-inflammatory, and antioxidant activities are recognized for some compounds isolated in this study. Compounds 6, 7, and 9 from *Phellinus linteus* (= *Sanghuangporus* sp.) have been reported for their antibacterial activity against oral pathogen *Porphyromonas gingivalis* [81]: compounds 6 and 7 showed a weak antibacterial activity (Minimum Inhibitory concentration, MIC: 34.1 and 155 μg/mL) while compound 9 showed a potent bacterial growth inhibition (MIC: 5.9 μg/mL, MIC of positive controls, hinokitiol and triclosan, was 25.0 and 3.13, respectively). Compound 7 was also reported to not only have antibacterial activity against *Micrococcus luteus* but also possess antifungal activities against *Mucor plumbeus* [12]. Compounds 3, 5, and 7 from the mycelium of *P. linteus* displayed hepatoprotective capability to hepatic fibrosis, conferring protection against it [11, 82]. Additionally, compound 7 exhibited antioxidant activities, albeit weaker than the effects of polyphenols [97].

AncA is well-preserved across *Basidiomycota* (Fig. 7). However, it does not belong to any known clades of STSs (I to IV) [98] and, thus, appears to synthesize a widely variant group of sesquiterpenoids compared to other mushrooms. It is worth noting that AncA ortholog was also found in peat moss (*Sphagnum* spp.). Based on the phylogenetic relationship, the *ancA* gene is presumed to have transferred over to *Sphagnum* spp. by horizontal gene transfer (HGT) from a bryophilous mushroom that is found in *Hymenochaetales* or from a species that is phylogenetically close to *Hymenochaetales*. The full complement of genes in the *ancA* BGC is not universally conserved across species (Supplementary Material 2: Fig. S3). This reflects species-specific adaptations in the biosynthetic capacity for sesquiterpenoids and the types of sesquiterpenoids produced, which may be shaped by various ecological factors. It may also explain why *Sanghuangporus* produces a particularly high number of medicinal compounds [99]. Regardless, the retention of a few key genes in *Hymenochaetaceae*, including the *ancA* gene (ACEPAI_7410) and nine other genes (ACEPAI_7414, ACEPAI_7419, ACEPAI_7420, ACEPAI_7421, ACEPAI_7434, ACEPAI_7435, ACEPAI_7438, ACEPAI_7439, and ACEPAI_7441), may suggest their essential role in the sesquiterpenoids biosynthetic pathway.

There are concerns regarding the future perspective of research on *Sanghuangporus.* First and foremost, *S. sanghuang* is still being reported by its old name. In addition, other *Sanghuangporus* species such as *S. baumii* and *S. vaninii* have been misleadingly reported as *sanghwang* (*S. sanghuang*) [100]. Similar issues were detected in this study, where phylogenomic and BCG comparative analyses suggest the misidentification of two reference genomic data (Fig. 6): GCA_001481415 *S. baumii* is assumed to be *S. sanghuang*, and GCA_009806525 *S. sanghuang* is assumed to be *S. vaninii* [31]. Additionally, although not utilized in this study due to the short genome assembly, GCA_016618145 *S. lonicericola* is assumed to be a *Trametes* species instead (Supplementary Material 2: Fig. S4). Misidentification and the use of wrong names significantly hinder the comprehensive understanding of the chemical profile for each *Sanghuangporus* species and urgently demand reidentification and subsequent reorganization of compounds [101].

## Conclusions

Genomic and metabolic data from four *Sanghuangporus* species, *S. baumii*, *S. sanghuang*, *S. vaninii*, and *S. weigelae*, were collectively analyzed to predict a BGC responsible for the biosynthesis of the isolated ABA-related sesquiterpenoids. Natural product genome mining was employed to find a BGC consisting a STS gene, *ancA*, that is universal across *Basidiomycota* and plays a pivotal role in the biosynthetic pathway of sesquiterpenoids. Further investigation into the correlation between ABA-related sesquiterpenoid profiles and the identified BGC holds promise for elucidating the intricate pathways involved in metabolite production. Future studies could focus on delineating the role of enzymes such as ACEPAI_7418 (a hypothetical protein), as well as exploring how different genetic components and gene orientations of *ancA* BGC across *Sanghuangporus* influence the metabolic types and concentrations. Integrating genetic techniques such as cloning, gene knock-out, and transcriptomics, along with compound feeding experiments, may provide valuable insights into the specific functions of these enzymes. Our findings represent an advancement toward fully understanding the medicinal potential of *Sanghuangporus* species.

## Supplementary Information

Supplementary Material 1.

Supplementary Material 2.

Supplementary Material 3.

## Data Availability

Genomic data and related information that support the findings in this study have been deposited in NCBI under the Bioproject PRJNA1153063.
