# Supplementary material for: A conserved terpene cyclase gene in Sanghuangporus for abscisic acid-related sesquiterpenoid biosynthesis
Source: BMC Genomics. 2025 Apr 15;26:378. doi: 10.1186/s12864-025-11542-9 (PMC12001456; doi:10.1186/s12864-025-11542-9)
Supplement: Supplementary file 1 — Supplementary Material 1. [file 12864_2025_11542_MOESM1_ESM.docx]

**Supplementary Method 1.** Identification of study materials

Small pieces (approximately 1×1 cm) of *Sanghuangporus* fruiting bodies and cultures on potato dextrose agar were isolated for the genomic DNA extraction using the AccuPrep Genomic DNA Extraction Kit (Bioneer, Daejeon, KR), following the manufacturer’s protocol. Polymerase chain reaction (PCR) was performed to amplify the internal transcribed spacer (ITS) region using the primers ITS1F and ITS4B (Gardes & Bruns, 1993). The PCR condition followed the initial denaturation at 95°C for 5 min; 35 cycles of 95°C for 40 s, 55°C for 40 s, and 72°C for 1 min; and a final extension at 72°C for 5 min. The PCR products were electrophoresed on 1% agarose gel using Gel Doc XR (Bio-Rad, CA, US) for verification and purified using the ExpinTM PCR Purifcation Kit (GeneAll Biotechnology, Seoul, KR) following the manufacturer’s instructions. Sequencing was performed using an ABI Prism 3700 Genetic Analyzer (Life Technologies, Gaithersburg, MD, US) at Macrogen (Seoul, KR) with the PCR primers. The obtained sequences were aligned with *Sanghuangporus* ITS sequences from type specimens and references using MAFFT version 7 (Katoh & Standley, 2013) in Geneious Prime 2024.0.3 (www.geneious.com). The ends of the alignments were manually trimmed. The maximum likelihood (ML) tree was inferred using the alignment data by RAxML v.8.2.12 (Stamatakis, 2014) with 1,000 replicates.

**Supplementary Method 2.** Modified CTAB protocol for genomic DNA extraction

Depending on the mycelial density, eight to twenty 1.5 ml tubes of dehydrated mycelia were prepared. They were ground using a Bead Ruptor Elite (OMNI International) in 500 μl of 2× cetyltrimethyl ammonium bromide (CTAB) buffer (Biosesang, Incheon, KR). Then, 20 µL Proteinase K (20 μg/mL) and 5 µL RNase solution (40 μg/mL; Bioneer, Daejeon, KR) were added and the mixture was left at 65°C for an hour. Phenol:chloroform:isoamyl alcohol (500 μl; 25:24:1, v/v; Invitrogen) was added and vortexed. The suspension was centrifuged (16.1 rcf, 20°C) for 15 min. Then, 300 μl of the supernatant was each transferred to a new 1.5 ml microtube, combining the contents of two tubes into one (i.e. 600 ml of supernatant per new tube). Per tube, 420 μl (70% volume of the supernatant) of iso-propylalcohol (Biosesang, Incheon, KR) was added, and after gentle inverting, the suspension was left at room temperature for 10 min. It was then centrifuged (16.1 rcf, 4°C) for 15 min. After removing the waste supernatant, DNA pellets were washed two times with 1 mL cold 70% (v/v) ethanol (Emsure, Darmstadt, Germany). During the washing steps, the DNA pellets were combined into one or two tubes using truncated 1000 μl tips. The pellets were air-dried, then dissolved in 20 to 50 μl of pure water and incubated at 65°C for an hour.

**Supplementary Method 3.** Electronic circular dichroism (ECD) calculation

Models of four diastereoisomers of compound **1** were built using the Chem3D software package, and stabilization of energy was performed by the Molecular Mechanic Force Field (MMFF), which is based on molecular mechanics. Conformational search was performed by the Maestro (version 9.9, Schrödinger LLC). For compound **1** six conformers for the 5S, 6S diastereomers were obtained using the Merck molecular force field (gas phase) with a 10 kJ/mol upper energy limit as the cutoff, and six conformers for the 5R, 6R diastereomers. The conformer with the highest calculated probability was selected. The selected conformers were optimized using the TurbomoleX 22.0 with the basis set def2-TZVPP for selected atoms and the functional B3-LYP were used to calculate ECD spectra. The calculated ECD spectra were compared with the experimental ECD spectrum (Nugroho et al., 2014).

**Supplementary Method 4.** Computational NMR chemical shift calculation for DP4+ analysis

Models for compound **1** were constructed using the Chem3D software suite, with energy minimization carried out based on the Molecular Mechanics Force Field (MMFF) approach. Conformers within a 10 kJ/mol energy threshold were further refined using density functional theory (DFT) at the B3LYP/6-31G (d,p) level. The shielding tensors for the optimized conformers were averaged according to Boltzmann weighting. Subsequent DP4+ analysis was conducted on these averaged tensors using a spreadsheet provided in the original study. Chemical shifts were determined from the previously reported GIAO magnetic shielding tensor values (Grimblat et al., 2015).

**References**

Gardes M, Bruns TD. ITS primers with enhanced specificity for basidiomycetes-application to the identification of mycorrhizae and rusts. Mol Ecol. 1993;2(2):113–118.

Grimblat N., Zanardi M. M., Sarotti A. M. Beyond DP4: an improved probability for the stereochemical assignment of isomeric compounds using quantum chemical calculations of NMR shifts. J Org Chem. 2015;80:12526–12534.

Katoh K, Standley DM. MAFFT multiple sequence alignment software version 7: improvements in performance and usability. Mol Biol Evol. 2013;30(4):772–780.

Nugroho, AE, Morita, H. Circular dichroism calculation for natural products. J. Nat. Med. 2014;68(1):1–10.

Stamatakis A. RAxML version 8: A tool for phylogenetic analysis and post-analysis of large phylogenies. Bioinformatics. 2014;30:1312–1313.
