# Supplementary material for: A conserved terpene cyclase gene in Sanghuangporus for abscisic acid-related sesquiterpenoid biosynthesis
Source: BMC Genomics. 2025 Apr 15;26:378. doi: 10.1186/s12864-025-11542-9 (PMC12001456; doi:10.1186/s12864-025-11542-9)
Supplement: Supplementary file 2 — Supplementary Material 2. [file 12864_2025_11542_MOESM2_ESM.pdf]

Supplementary Figure 1. Maximum likelihood phylogenetic tree (RAxML, 1,000 bootstrap iterations) of *Sanghuangporus* based on ITS. Sequences of *Inonotus henanensis* (Dai 12221) and *I. griseus* (Dai 13436) were used as outgroup. Bootstrap values over 70% are indicated. TYPE refers to sequence of a type material and REF refers to sequence of a reference specimen from other studies.

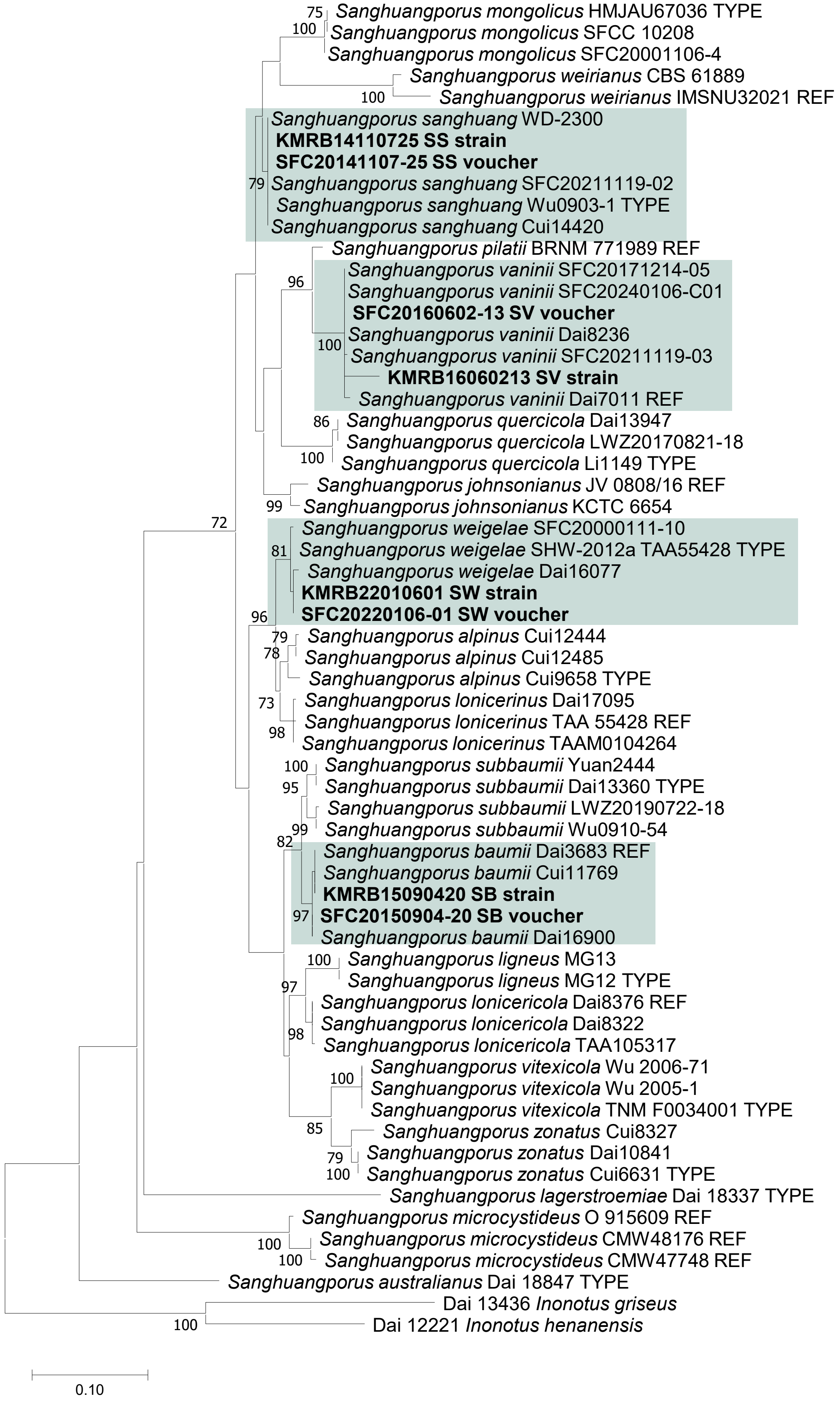

Supplementary Figure 2. Maximum likelihood phylogenetic tree (RAxML, 1,000 bootstrap iterations) of top 100 NCBI blastp hits of *Sanghuangporus* AncA amino acid sequences. Sequence of *Geoglossum umbratile* M1839_002554 (KAI9772142) was used as an outgroup. Node bootstrap support values ≥70 are indicated.

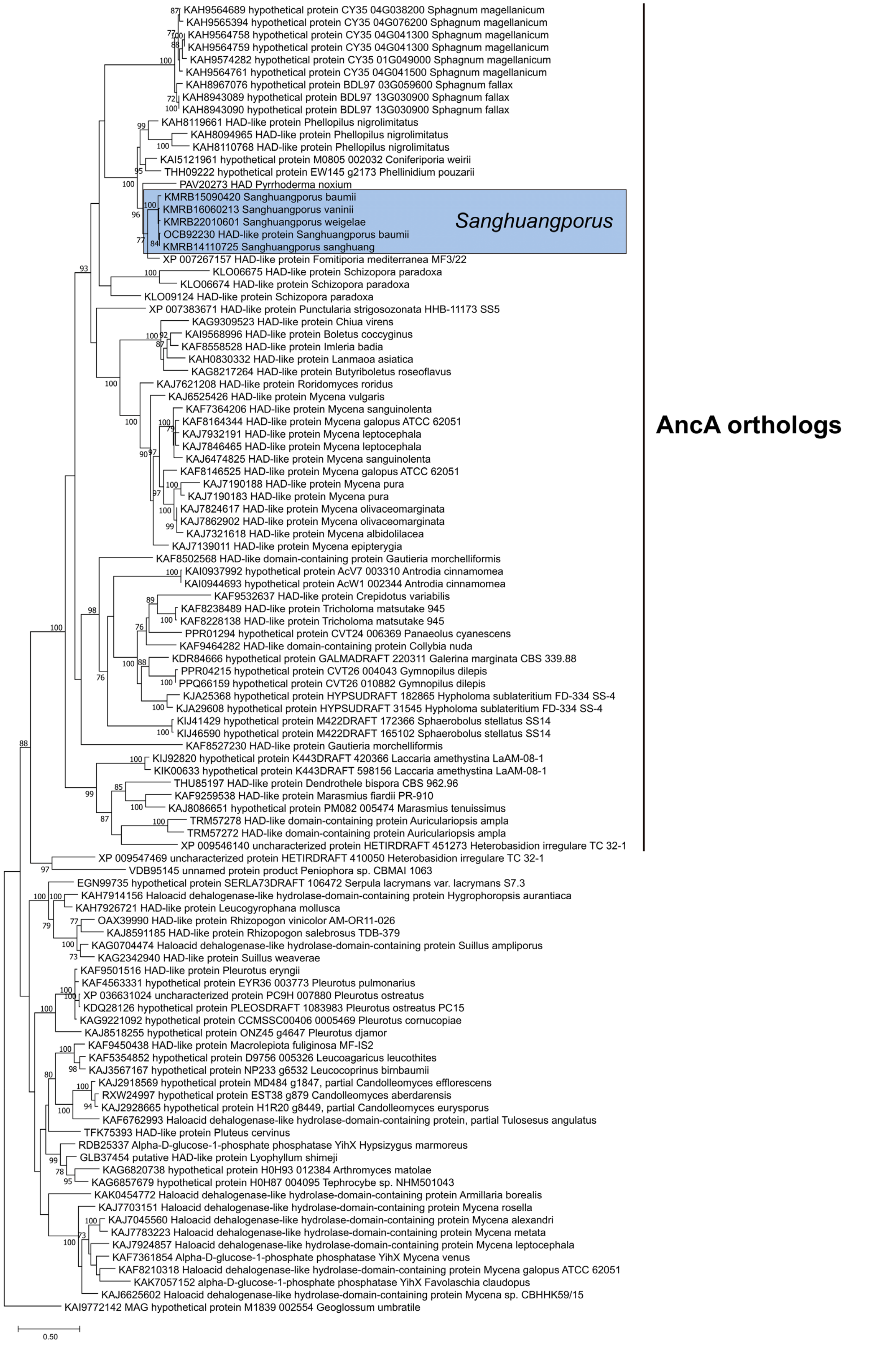

Supplementary Figure 3. Comparative synteny analysis of *ancA* BGC across *Hymenochaetaceae* family. A phylogenomic species tree of *Hymenochaetaceae* is given on the left. The blue boxes follow the order of genes in the BGC. The numbers in the box indicate the copy number of each gene, and the color intensity corresponds to the similarity of each gene with that of *Sanghuangporus weigelae* (reference species).

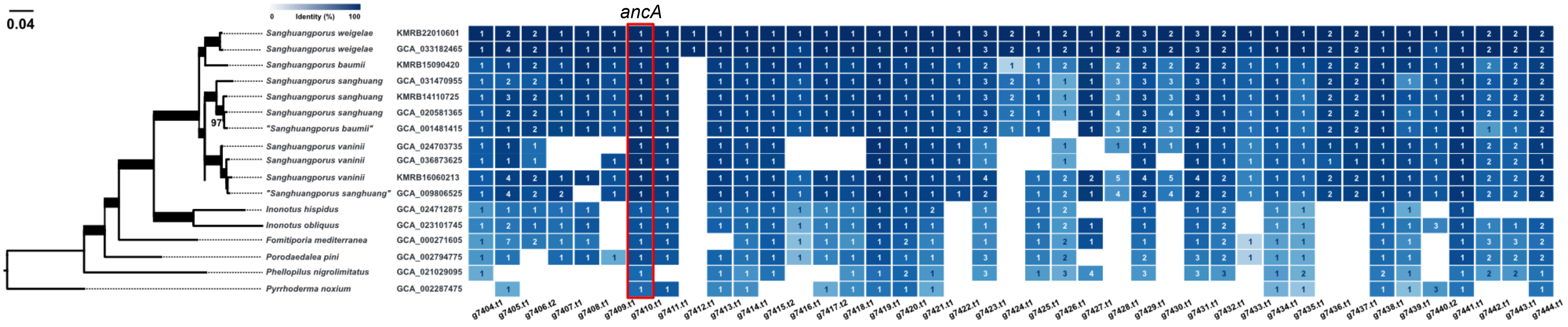

Supplementary Figure 4. Top 50 hits of NCBI BLASTn result of GCA_016618145 *Sanghuangporus lonicericola* partial large subunit ribosomal RNA gene (nLSU). The nLSU region has been analyzed instead of the fungal universal barcode, internal transcribed spacer regions, to avoid a possible misperception (Shen et al., 2021, https://doi.org/10.1186/s43008-021-00059-x).

Sequences producing significant alignments Download Select columns Show 50

select all *50 sequences selected* GenBank Graphics Distance tree of results MSA Viewer

Description	Scientific Name	Max Score	Total Score	Query Cover	E value	Per. Ident	Acc. Len	Accession
Trametes hirsuta voucher LWZ 20210925-13a large subunit ribosomal RNA gene, partial sequence	Trametes hirsuta	1048	1048	100%	0.0	100.00%	852	ON885423.1
Trametes hirsuta gene for 28S ribosomal RNA, partial sequence, strain: KU-RNB018	Trametes hirsuta	1048	1048	100%	0.0	100.00%	1341	LC190471.1
Trametes hirsuta isolate KMRB18100832 large subunit ribosomal RNA gene, partial sequence	Trametes hirsuta	1048	1048	100%	0.0	100.00%	1331	ON402839.1
Trametes hirsuta isolate SYLH-1 large subunit ribosomal RNA gene, partial sequence	Trametes hirsuta	1048	1048	100%	0.0	100.00%	1057	ON139203.1
Trametes sp. strain WML2020-100 large subunit ribosomal RNA gene, partial sequence	Trametes sp.	1048	1048	100%	0.0	100.00%	974	OM065725.1
Trametes hirsuta isolate CONTIG_SL27 large subunit ribosomal RNA gene, partial sequence	Trametes hirsuta	1048	1048	100%	0.0	100.00%	1266	OL688363.1
Trametes hirsuta voucher Dai12319 25S large subunit ribosomal RNA gene, partial sequence	Trametes hirsuta	1048	1048	100%	0.0	100.00%	1350	KC848383.1
Trametes hirsuta voucher Cui7784 25S large subunit ribosomal RNA gene, partial sequence	Trametes hirsuta	1048	1048	100%	0.0	100.00%	1349	KC848382.1
Trametes hirsuta voucher Cui 7784 large subunit ribosomal RNA gene, partial sequence	Trametes hirsuta	1048	1048	100%	0.0	100.00%	1402	JN048787.1
Trametes hirsuta strain Wu 9410-39 28S ribosomal RNA gene, partial sequence	Trametes hirsuta	1048	1048	100%	0.0	100.00%	938	AY351922.1
Trametes versicolor partial nuclear 25S ribosomal RNA (25S rRNA gene), GEL5033	Trametes versic...	1048	1048	100%	0.0	100.00%	913	AJ406538.1
Trametes hirsuta culture CBS:282.73 strain CBS 282.73 large subunit ribosomal RNA gene, partial sequence	Trametes hirsuta	1042	1042	100%	0.0	99.82%	908	MH872390.1
Ceriporia tarda culture CBS:449.48 strain CBS 449.48 large subunit ribosomal RNA gene, partial sequence	Ceriporia tarda	1042	1042	100%	0.0	99.82%	943	MH867975.1
Trametes hirsuta culture CBS:248.30 strain CBS 248.30 large subunit ribosomal RNA gene, partial sequence	Trametes hirsuta	1042	1042	100%	0.0	99.82%	931	MH866579.1
Trametes hirsuta culture CBS:320.29 strain CBS 320.29 large subunit ribosomal RNA gene, partial sequence	Trametes hirsuta	1042	1042	100%	0.0	99.82%	938	MH866536.1
Trametes hirsuta voucher Cui 7720 28S ribosomal RNA gene, partial sequence	Trametes hirsuta	1042	1042	100%	0.0	99.82%	1337	KX880681.1
Trametes hirsuta voucher HMUT 1172 large subunit ribosomal RNA gene, partial sequence	Trametes hirsuta	1042	1042	100%	0.0	99.82%	1036	OM914763.1
Trametes hirsuta voucher HMUT 915 large subunit ribosomal RNA gene, partial sequence	Trametes hirsuta	1042	1042	100%	0.0	99.82%	1028	OM914762.1
Trametes hirsuta voucher HMUT 878 large subunit ribosomal RNA gene, partial sequence	Trametes hirsuta	1042	1042	100%	0.0	99.82%	1041	OM914761.1
Trametes hirsuta voucher HMUT 749 large subunit ribosomal RNA gene, partial sequence	Trametes hirsuta	1042	1042	100%	0.0	99.82%	1045	OM914760.1
Trametes hirsuta strain DMC819 28S ribosomal RNA gene, partial sequence	Trametes hirsuta	1042	1042	100%	0.0	99.82%	940	KC589168.1
Trametes hirsuta voucher F445 large subunit ribosomal RNA gene, partial sequence	Trametes hirsuta	1042	1042	100%	0.0	99.82%	932	OR602385.1
Trametes hirsuta isolate SJY-4 large subunit ribosomal RNA gene, partial sequence	Trametes hirsuta	1042	1042	100%	0.0	99.82%	1013	OQ165179.1
Trametes hirsuta isolate SYLH-1 large subunit ribosomal RNA gene, partial sequence	Trametes hirsuta	1042	1042	100%	0.0	99.82%	1029	OQ165178.1
Trametes tephroleuca voucher Cui 16200 large subunit ribosomal RNA gene, partial sequence	Trametes tephr...	1042	1042	100%	0.0	99.82%	1343	OK642269.1
Trametes hirsuta isolate KMRB17121406 large subunit ribosomal RNA gene, partial sequence	Trametes hirsuta	1042	1042	100%	0.0	99.82%	1344	ON402840.1
Trametes hirsuta isolate KMRB18101711 large subunit ribosomal RNA gene, partial sequence	Trametes hirsuta	1042	1042	100%	0.0	99.82%	1344	ON402838.1
Trametes hirsuta isolate SJY-4 large subunit ribosomal RNA gene, partial sequence	Trametes hirsuta	1042	1042	100%	0.0	99.82%	1020	ON139208.1
Trametes tephroleuca voucher Cui7987 25S large subunit ribosomal RNA gene, partial sequence	Trametes tephr...	1042	1042	100%	0.0	99.82%	1349	KC848378.1
Trametes pubescens voucher Cui7569 25S large subunit ribosomal RNA gene, partial sequence	Trametes pubes...	1042	1042	100%	0.0	99.82%	1350	KC848377.1
Trametes menziesii voucher Dai6782 25S large subunit ribosomal RNA gene, partial sequence	Trametes menzi...	1042	1042	100%	0.0	99.82%	1350	KC848374.1
Trametes sp. HJL-2013b voucher Yuan3451 25S large subunit ribosomal RNA gene, partial sequence	Trametes ellips...	1042	1042	100%	0.0	99.82%	1350	KC848344.1
Trametes ellipsoidea BJFC Yuan 3453 28S rRNA gene, partial sequence; from TYPE material	Trametes ellips...	1042	1042	100%	0.0	99.82%	1350	NG_075180.1
Trametes sp. strain M424 large subunit ribosomal RNA gene, partial sequence	Trametes sp.	1042	1042	100%	0.0	99.82%	931	MW520044.1
Trametes hirsuta voucher YG/Ch40 large subunit ribosomal RNA gene, partial sequence	Trametes hirsuta	1042	1042	100%	0.0	99.82%	862	MT524614.1
Trametes hirsuta voucher YG/PS168 large subunit ribosomal RNA gene, partial sequence	Trametes hirsuta	1042	1042	100%	0.0	99.82%	861	MT524613.1
Trametes hirsuta voucher YG/PS128 large subunit ribosomal RNA gene, partial sequence	Trametes hirsuta	1042	1042	100%	0.0	99.82%	861	MT524612.1
Trametes hirsuta voucher YG314 large subunit ribosomal RNA gene, partial sequence	Trametes hirsuta	1042	1042	100%	0.0	99.82%	861	MT524611.1
Trametes hirsuta voucher RLG5133T 25S ribosomal RNA gene, partial sequence	Trametes hirsuta	1042	1042	100%	0.0	99.82%	1351	JN164801.1
Trametes hirsuta strain GLMC 467 large subunit ribosomal RNA gene, partial sequence	Trametes hirsuta	1042	1042	100%	0.0	99.82%	877	MT156314.1
Trametes hirsuta voucher ZRL2015001_1 28S ribosomal RNA gene, partial sequence	Trametes hirsuta	1038	1038	100%	0.0	99.65%	931	KY418904.1
Trametes hirsuta strain DMC716 28S ribosomal RNA gene, partial sequence	Trametes hirsuta	1037	1037	100%	0.0	99.65%	932	KC589167.1
Trametes hirsuta strain DMC341 28S ribosomal RNA gene, partial sequence	Trametes hirsuta	1037	1037	100%	0.0	99.65%	934	KC589166.1
Trametes hirsuta strain DMC333 28S ribosomal RNA gene, partial sequence	Trametes hirsuta	1037	1037	100%	0.0	99.65%	937	KC589165.1
Trametes hirsuta isolate SJY-6 large subunit ribosomal RNA gene, partial sequence	Trametes hirsuta	1037	1037	100%	0.0	99.65%	1020	OQ165180.1
Trametes hirsuta isolate SJY-6 large subunit ribosomal RNA gene, partial sequence	Trametes hirsuta	1037	1037	100%	0.0	99.65%	1052	ON139210.1
Trametes hirsuta voucher QHU20252 large subunit ribosomal RNA gene, partial sequence	Trametes hirsuta	1037	1037	100%	0.0	99.65%	1397	OM942721.1
Trametes hirsuta isolate Z4 large subunit ribosomal RNA gene, partial sequence	Trametes hirsuta	1037	1037	100%	0.0	99.65%	1388	OL960195.1
Trametes villosa culture CBS:334.49 strain CBS 334.49 large subunit ribosomal RNA gene, partial sequence	Trametes villosa	1031	1031	100%	0.0	99.47%	858	MH868069.1
Trametes sp. isolate (V63) large subunit ribosomal RNA gene, partial sequence	Trametes sp.	1031	1031	100%	0.0	99.47%	944	MW553719.1

Supplementary Figure 5. ^{1}H, 13C spectrum of SHA-1 ($CDCl_3$ 600MHz).

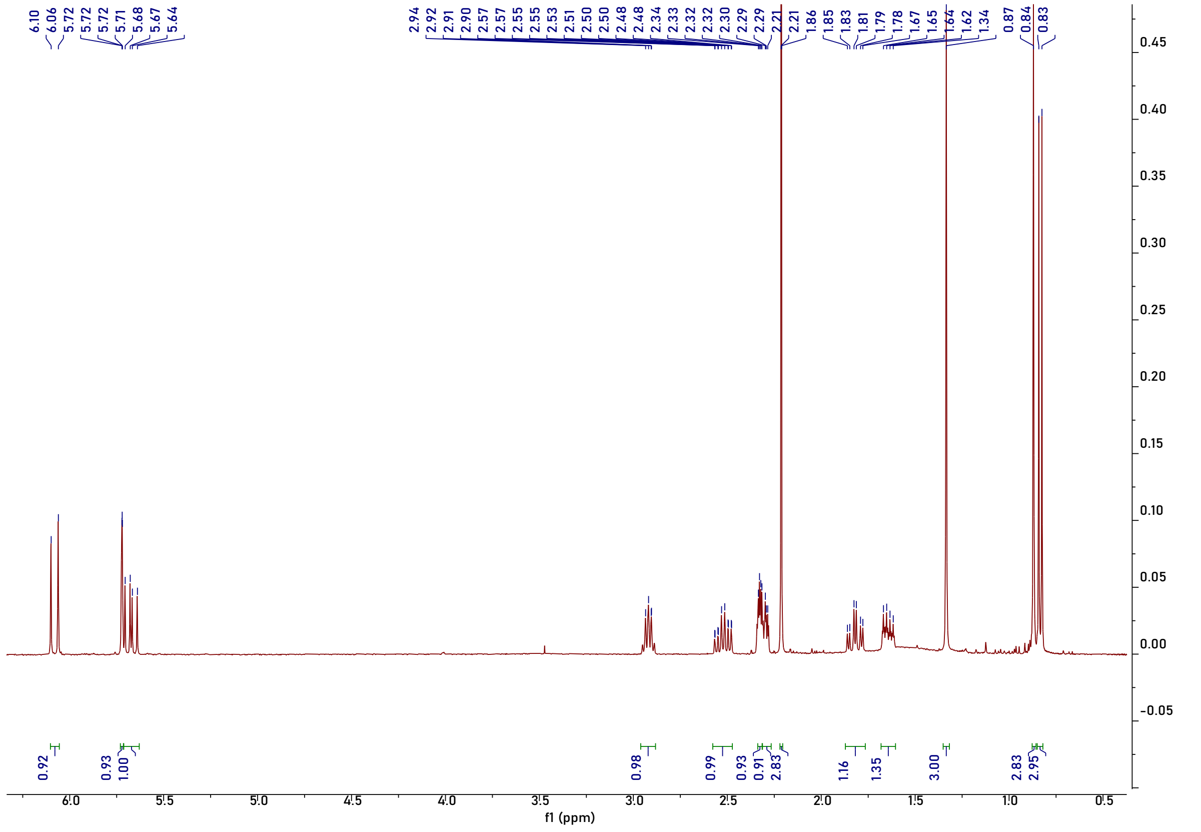

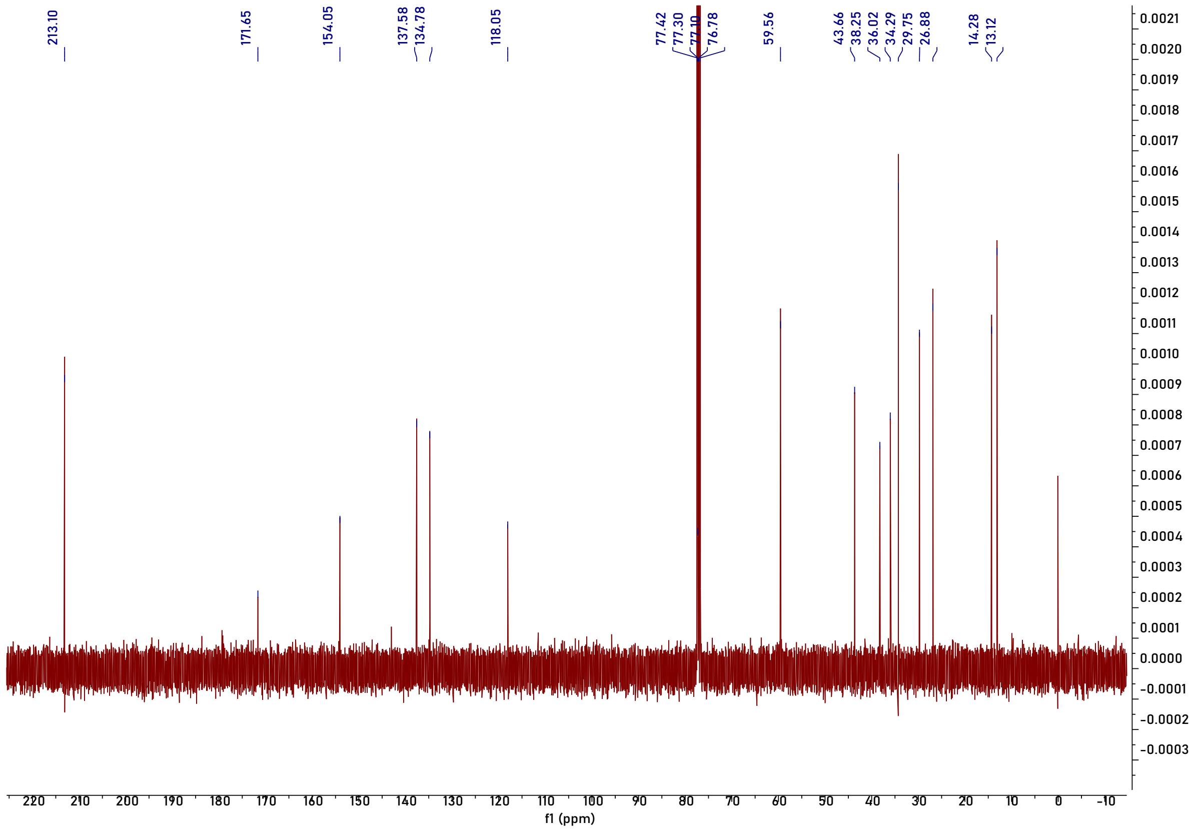

Supplementary Figure 6. COSY, HMBC spectrum of SHA-1 ($CDCl_3$, 600MHz).

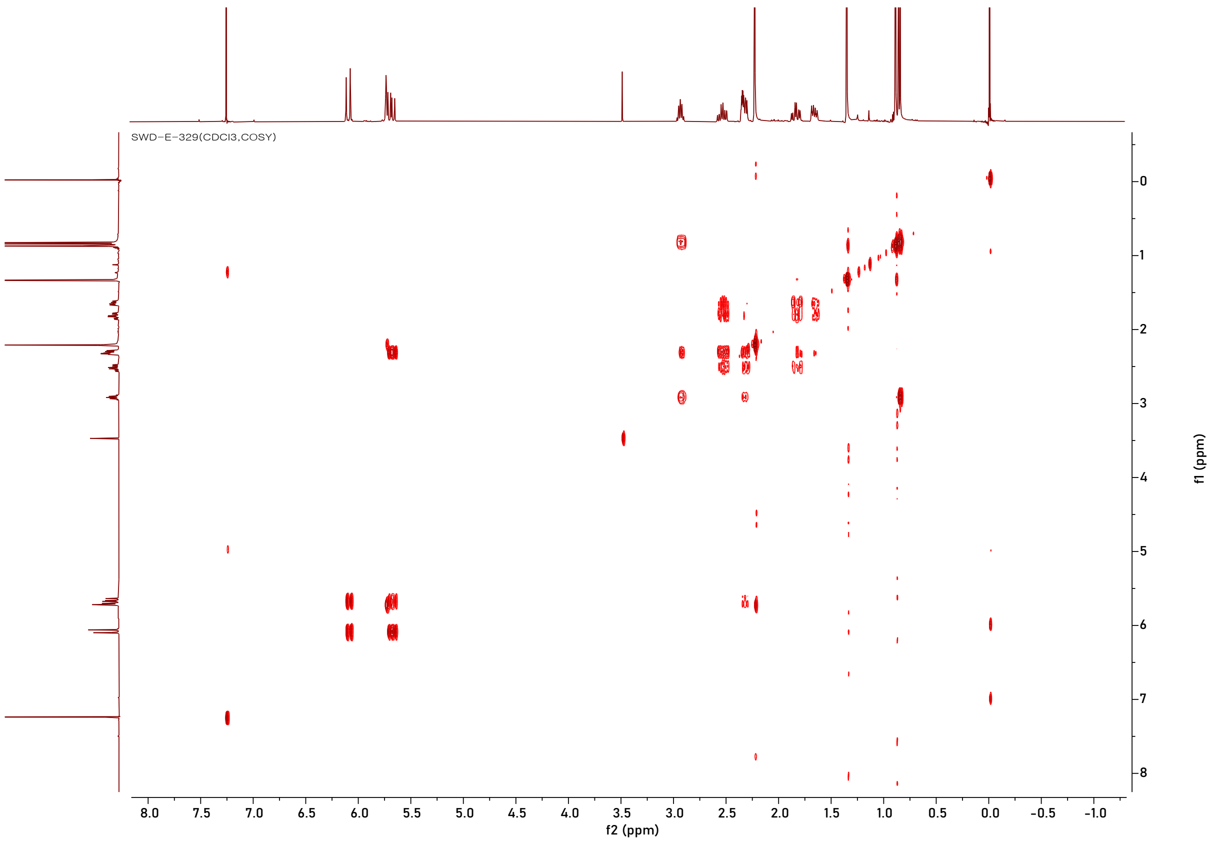

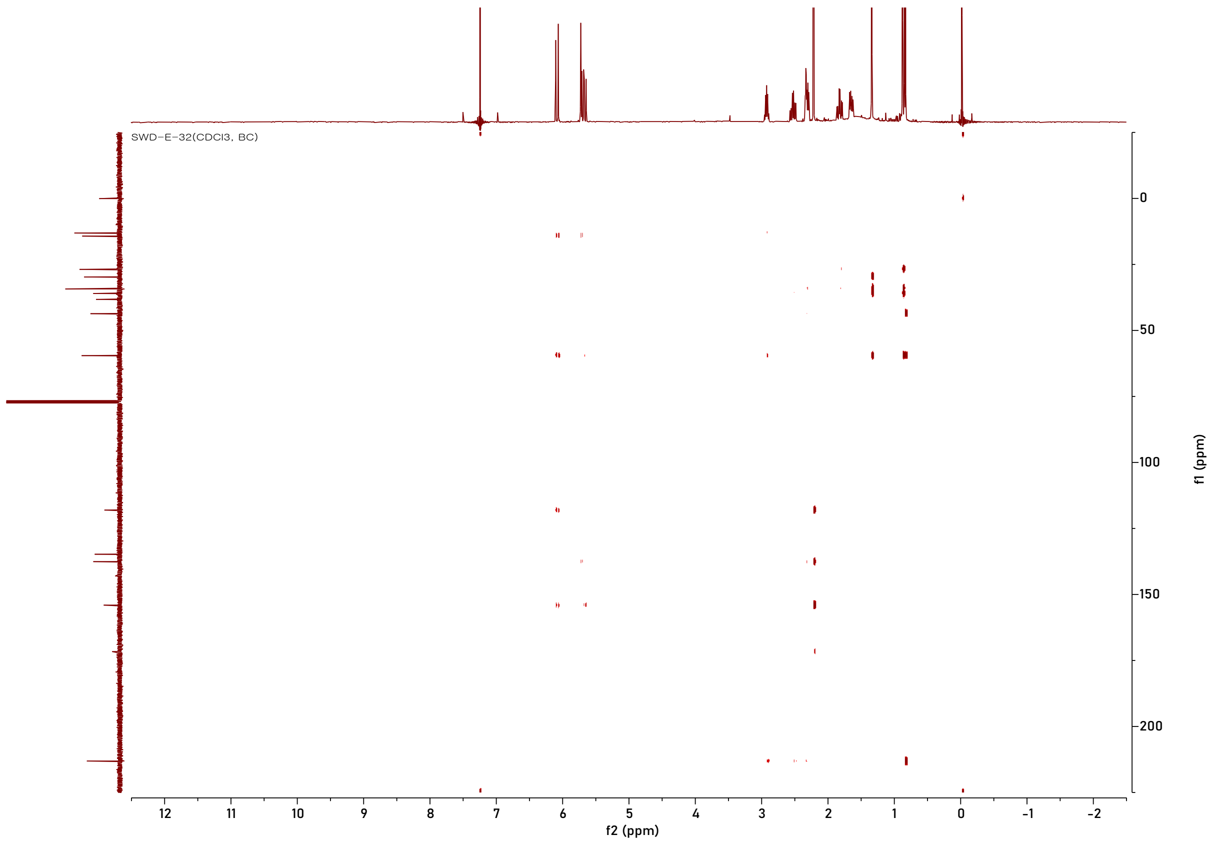

Supplementary Figure 7. HSQC, NOESY spectrum of SHA-1 ($CDCl_3$, 600MHz).

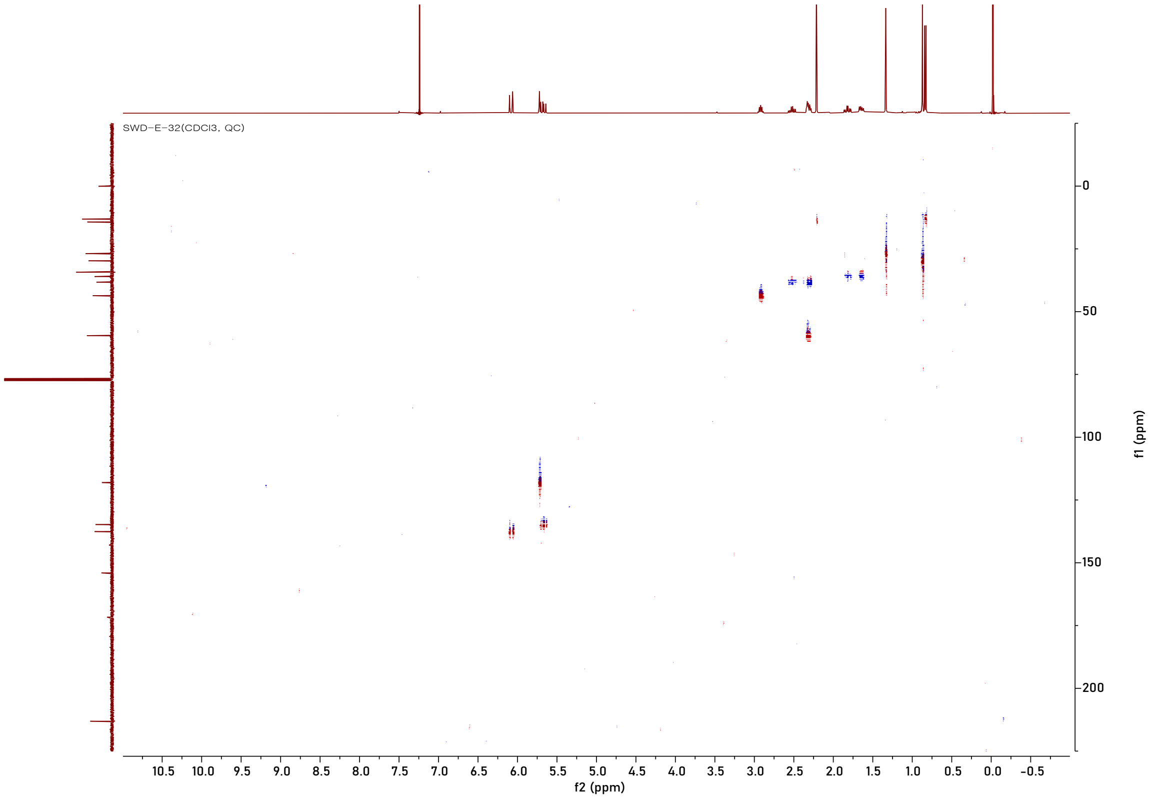

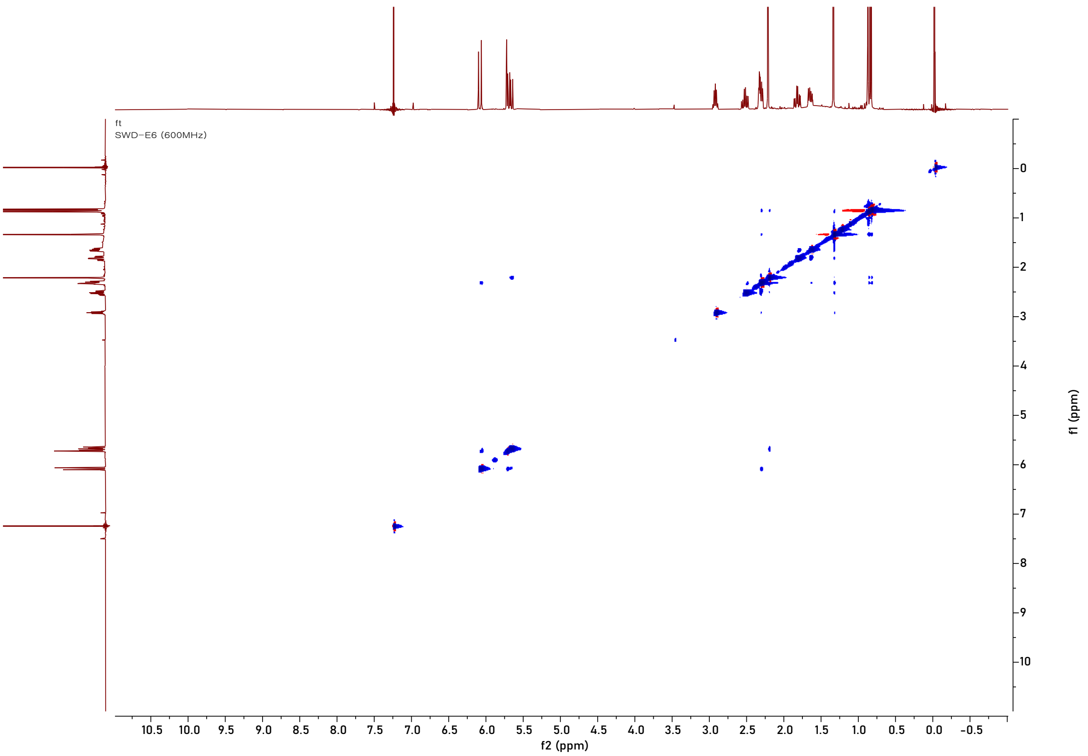

Supplementary Figure 8. HR-ESI-MS spectrum data of SHA-1.

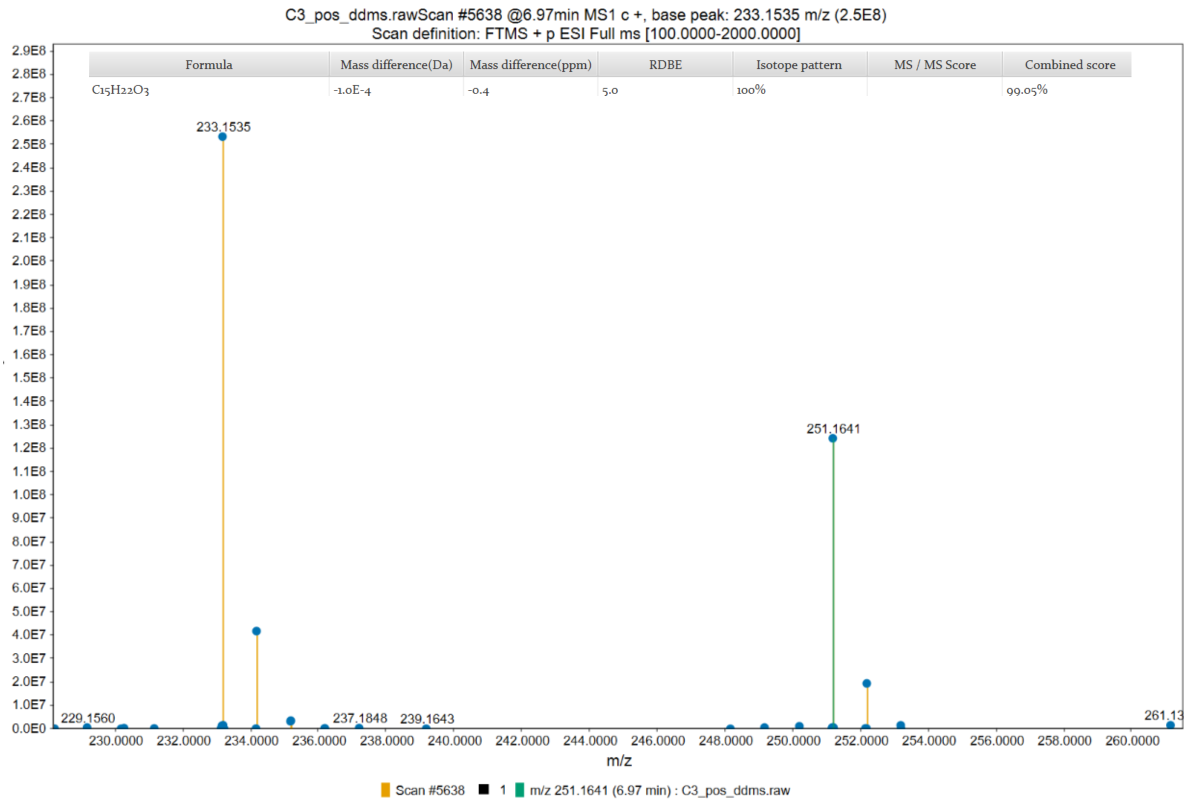

Supplementary Figure 9. ^{1}H NMR data of compounds **2-4** ($CDCl_3$, 400MHz).

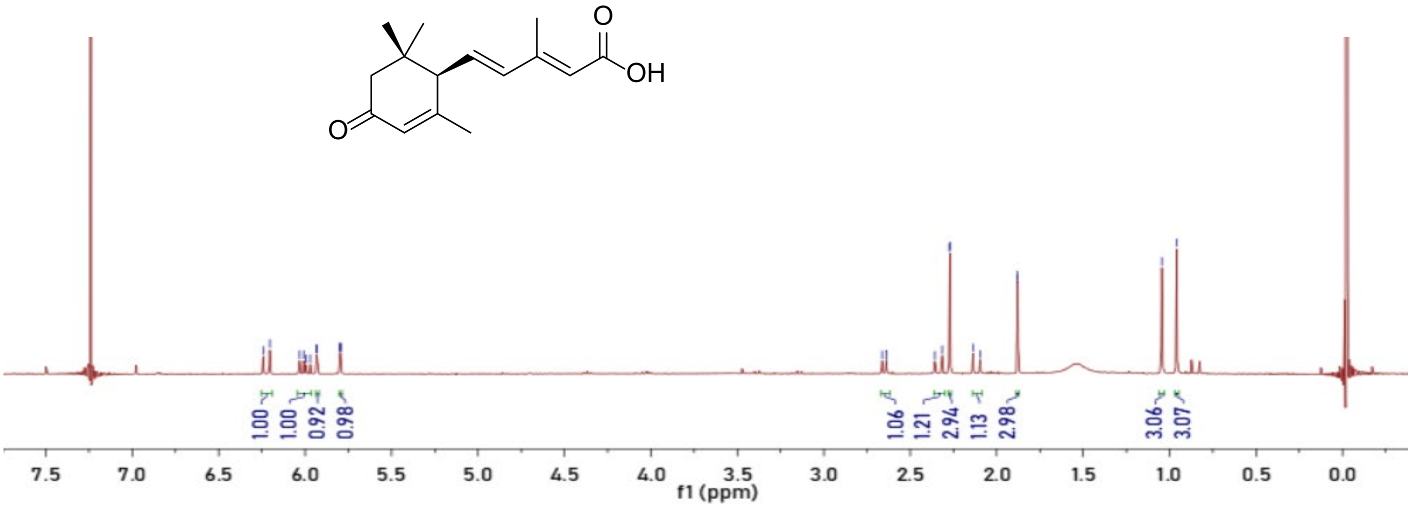

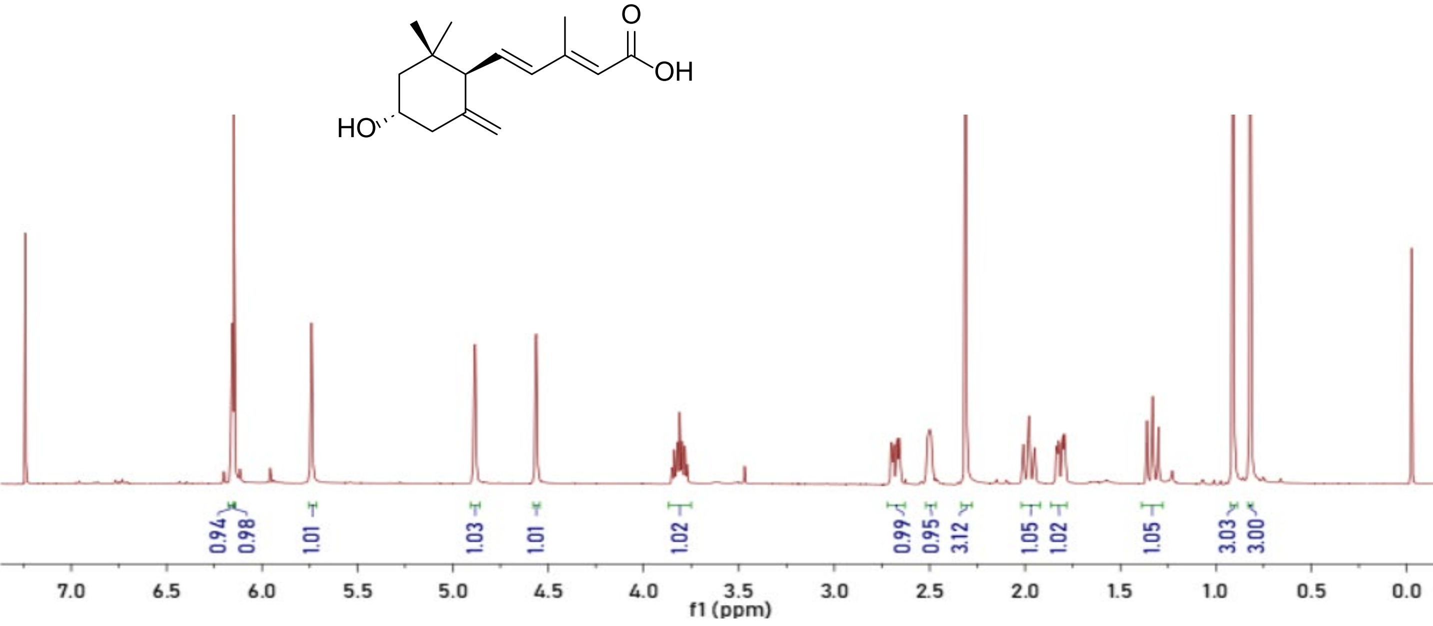

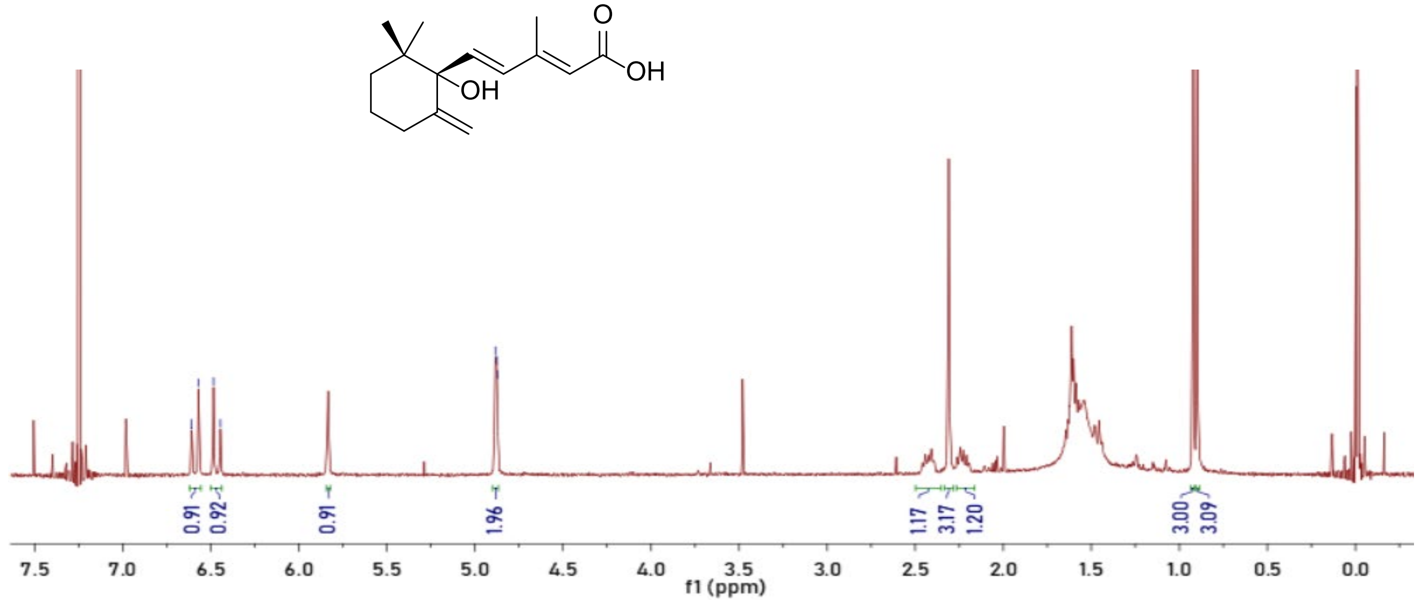

Supplementary Figure 10. ^{1}H NMR data of compounds **4-7**.

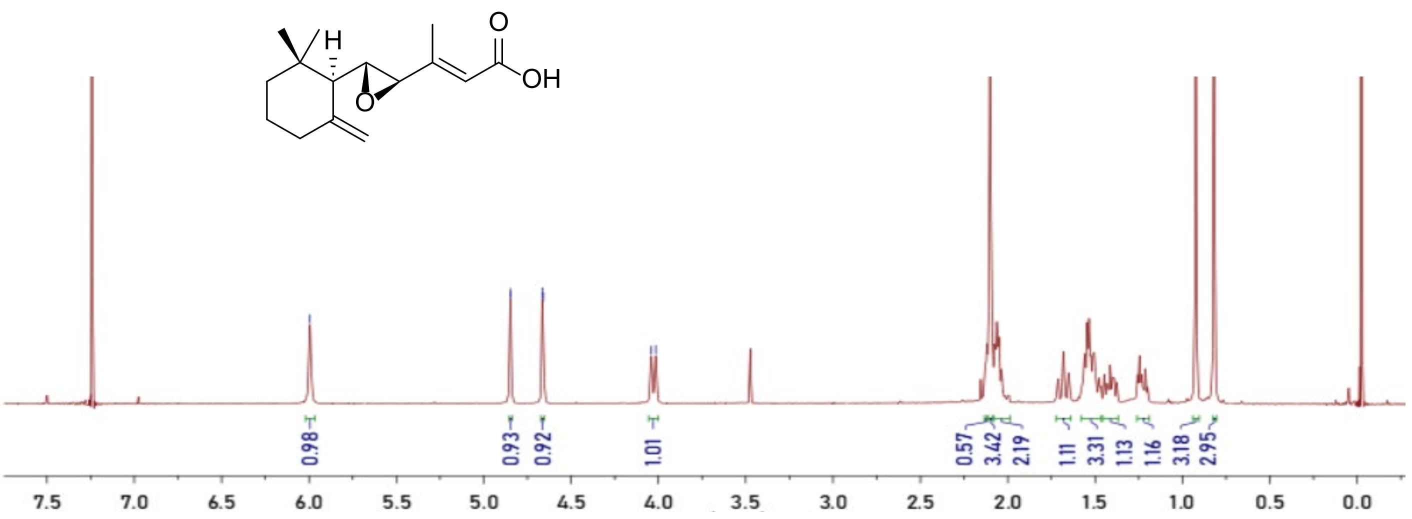

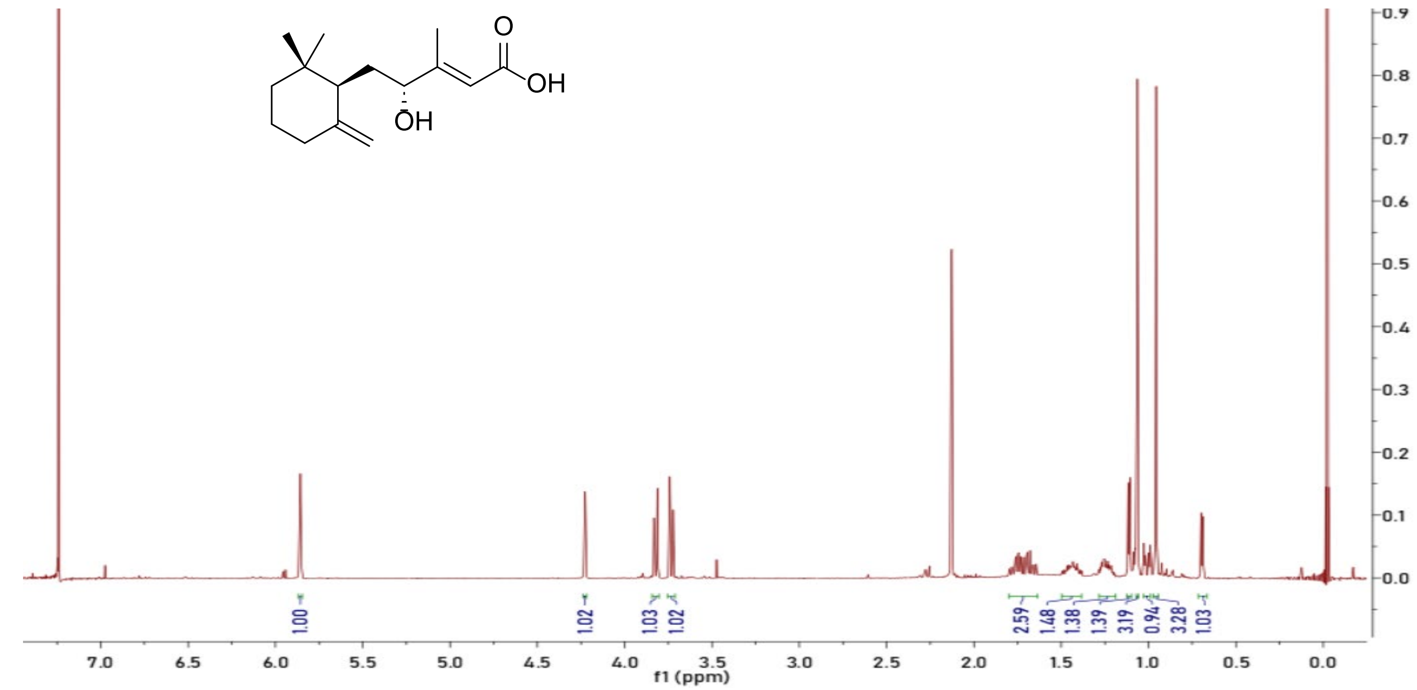

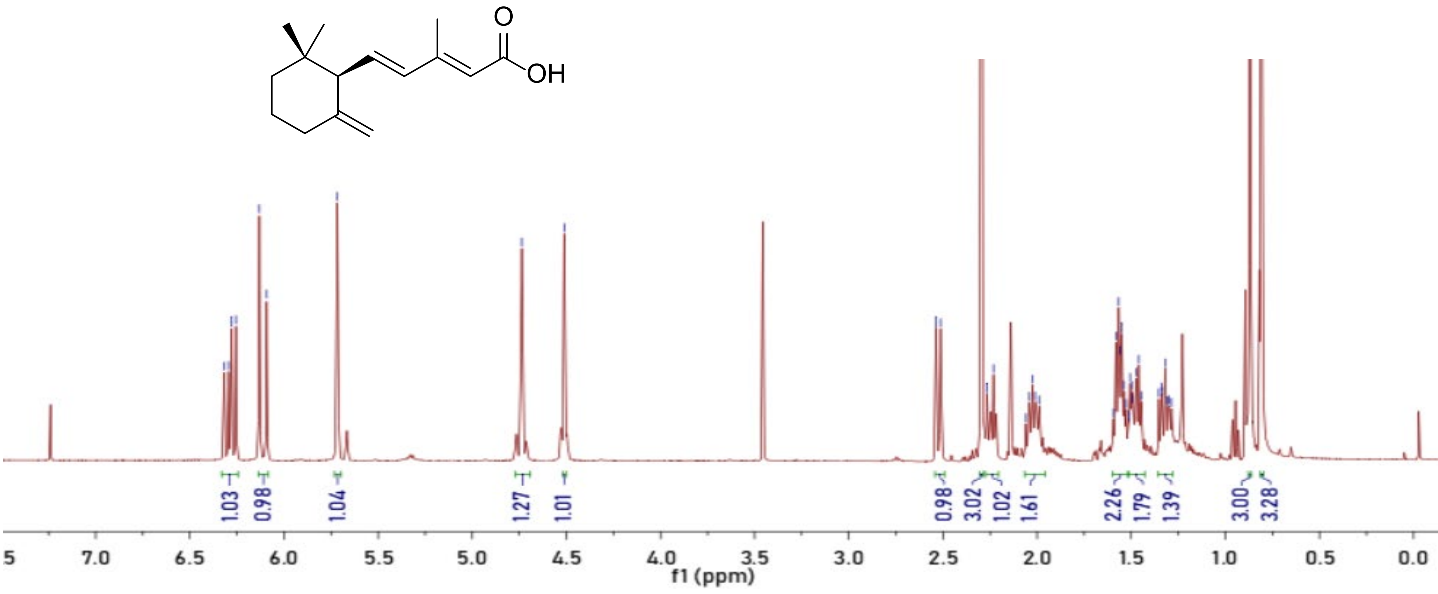

Supplementary Figure 11. Energy optimized conformers and energy analysis data of SHA-1.

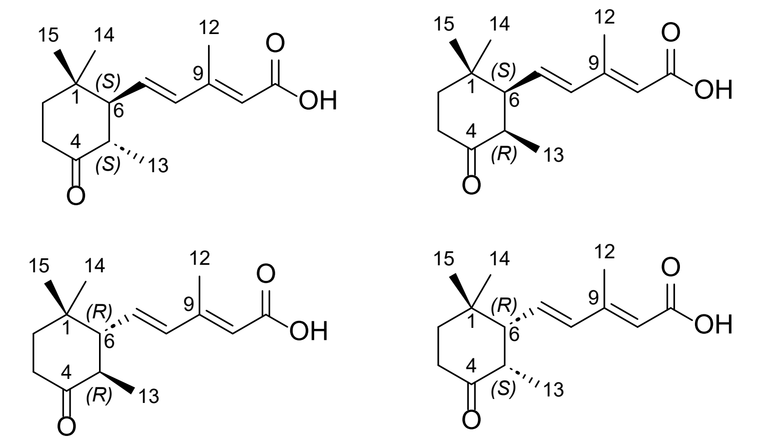

**Row**	**Title**	**Potential energy**	**Relative potential energy**	**Boltzmann population**
5S6R	1	27.702	0.000	66.527678
	2	31.898	4.196	12.239923
	3	31.979	4.277	11.847039
	4	35.063	7.361	3.414950
	5	35.208	7.506	3.220171
	6	35.599	7.897	2.750239
5R6S	1	27.702	0.000	66.527678
	2	31.898	4.196	12.239923
	3	31.979	4.277	11.847039
	4	35.063	7.361	3.414950
	5	35.208	7.506	3.220171
	6	35.599	7.897	2.750239
5R6S	1	32.284	0.000	63.677971
	2	35.721	3.437	15.917889
	3	36.522	4.238	11.520870
	4	39.183	6.899	3.937570
	5	39.409	7.125	3.594399
	6	41.834	9.550	1.351300
5R6S	1	27.702	0.000	66.527678
	2	31.898	4.196	12.239923
	3	31.979	4.277	11.847039
	4	35.063	7.361	3.414950
	5	35.208	7.506	3.220171
	6	35.599	7.897	2.750239

Supplementary Figure 12. Quantum mechanics-based computational analysis of SHA-1 using DP4+ statistical calculations.

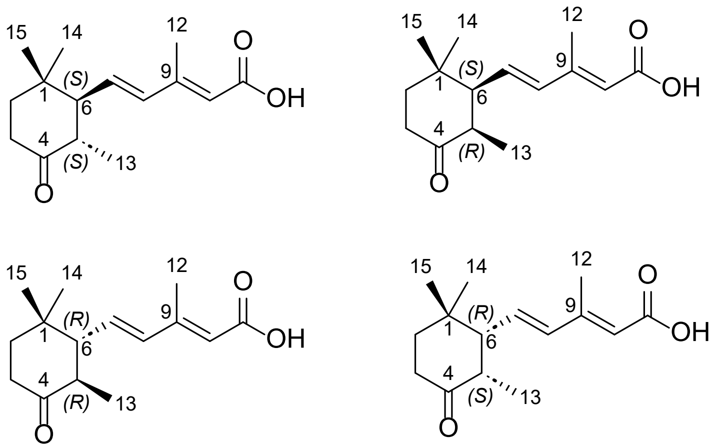

	5S6S	5R6S	5R6R	5R6S
DP4+ (H data)	100.00%	0.00%	0.00%	0.00%
DP4+ (C data)	99.31%	0.00%	0.69%	0.00%
DP4+ (all data)	100.00%	0.00%	0.00%	0.00%

N.O.	Experimental	5S6S*	5R6S*	5R6R*	5R6S*
C-1	34.3	39.1	39.3	39.2	39.3
C-2	36	40.7	46.6	40.5	46.7
C-3	38.3	41.3	41.2	41.1	41
C-4	213.1	215.5	214.3	215.5	214.2
C-5	43.7	46.5	47.1	46.8	46.8
C-6	59.6	64.1	64.8	64.4	65
C-7	134.8	137.7	139.1	138.6	140
C-8	137.6	139.4	139	139.5	139
C-9	154.05	154.08	154.21	153.57	153.63
C-10	118.05	118.99	118.68	119.06	119.18
C-11	171.65	161.86	161.75	161.8	161.76
C-12	14.28	18.64	19.12	18.34	18.7
C-13	13.12	21.23	21.10	21.37	21.13
C-14	29.8	36.7	36.7	33.2	26.3
C-15	26.4	33	26.3	36.3	36.8
H-2a	1.64	1.22	1.32	1.45	1.24
H-2b	1.82	1.41	1.26	1.2	1.29
H-4a	2.29	1.72	1.75	1.92	1.93
H-4b	2.52	1.98	1.93	1.78	1.78
H-5	2.92	2.32	1.91	2.34	1.9
H-6	2.32	1.94	1.52	1.88	1.52
H-7	5.67	5.29	5.53	5.44	5.54
H-8	6.08	5.63	5.53	5.58	5.58
H-10	5.72	4.86	4.98	4.91	4.95
H-12	2.21	1.73	1.79	1.73	1.8
H-13	0.83	0.53	0.59	0.55	0.57
H-14	0.87	0.61	0.61	0.97	0.82
H-15	1.34	0.96	0.84	0.59	0.62

*DP4 calculated data

Supplementary Figure 13. Atom coordination data of ECD calculated conformers

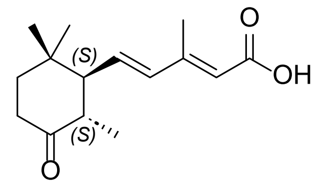

N.O	Atom	X	Y	Z
1	C	-11.4966	-0.6941	-2.4806
2	C	-11.4341	-2.2153	-2.4186
3	C	-10.7968	-2.6838	-1.1298
4	C	-9.4518	-2.0585	-0.775
5	C	-9.556	-0.5105	-0.8152
6	C	-10.1428	-0.003	-2.1829
7	O	-11.3097	-3.5833	-0.4652
8	C	-10.3638	1.5273	-2.1641
9	C	-9.1446	-0.2876	-3.3305
10	C	-10.3377	0.0128	0.3832
11	C	-9.82	0.8543	1.2949
12	C	-10.507	1.3947	2.4718
13	C	-9.8418	2.2842	3.2374
14	C	-10.3469	2.9747	4.4375
15	O	-9.3334	3.6238	5.0383
16	O	-11.476	3.0183	4.88
17	C	-11.9062	0.9011	2.7459
18	C	-8.9096	-2.5958	0.5517
19	H	-11.8639	-0.3942	-3.4703
20	H	-12.2509	-0.348	-1.7619
21	H	-12.4485	-2.6246	-2.4806
22	H	-10.8618	-2.6291	-3.2549
23	H	-8.749	-2.3911	-1.5487
24	H	-8.5292	-0.1226	-0.7394
25	H	-10.6845	1.8904	-3.1477
26	H	-9.4404	2.0552	-1.9004
27	H	-11.1392	1.8229	-1.4494
28	H	-9.5277	0.0904	-4.2857
29	H	-8.9445	-1.3534	-3.4676
30	H	-8.1839	0.2055	-3.1429
31	H	-11.3632	-0.3362	0.4808
32	H	-8.7893	1.1814	1.1536
33	H	-8.8249	2.5695	2.9809
34	H	-9.7601	4.0465	5.8128
35	H	-12.3132	1.2268	3.7033
36	H	-12.5925	1.2465	1.965
37	H	-11.9286	-0.1945	2.7733
38	H	-7.9611	-2.1136	0.81
39	H	-8.7271	-3.674	0.4801
40	H	-9.6129	-2.4458	1.3777

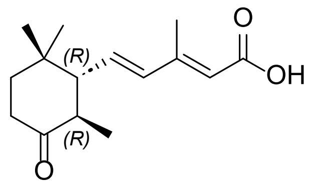

N.O	Atom	X	Y	Z
1	C	-11.2023	-2.7046	-2.739
2	C	-12.0219	-2.8959	-1.4687
3	C	-11.1699	-3.4445	-0.3464
4	C	-9.8691	-2.7015	-0.0605
5	C	-9.0428	-2.5511	-1.3656
6	C	-9.893	-1.9063	-2.5206
7	O	-11.5653	-4.3851	0.3414
8	C	-10.2264	-0.4342	-2.18
9	C	-9.1035	-1.8804	-3.8499
10	C	-8.423	-3.882	-1.7729
11	C	-7.0978	-4.0783	-1.8852
12	C	-6.4306	-5.3267	-2.2662
13	C	-5.0876	-5.3111	-2.3925
14	C	-4.221	-6.4331	-2.7956
15	O	-2.9333	-6.0906	-2.6116
16	O	-4.5287	-7.5182	-3.2435
17	C	-7.2997	-6.5423	-2.4744
18	C	-9.085	-3.3511	1.0824
19	H	-10.9642	-3.694	-3.1509
20	H	-11.8264	-2.2072	-3.4923
21	H	-12.4685	-1.9549	-1.1324
22	H	-12.8411	-3.5962	-1.6656
23	H	-10.1603	-1.7062	0.2966
24	H	-8.2211	-1.8549	-1.141
25	H	-10.7921	0.0373	-2.9922
26	H	-9.3102	0.1496	-2.0351
27	H	-10.8272	-0.3316	-1.2727
28	H	-8.1404	-1.3726	-3.7259
29	H	-9.662	-1.3477	-4.6286
30	H	-8.9087	-2.8877	-4.233
31	H	-9.1155	-4.6971	-1.9702
32	H	-6.442	-3.2308	-1.6824
33	H	-4.5338	-4.3956	-2.2016
34	H	-2.4415	-6.8865	-2.9034
35	H	-6.7425	-7.4683	-2.6179
36	H	-7.9364	-6.7139	-1.5988
37	H	-7.9403	-6.4075	-3.3528
38	H	-8.1386	-2.8278	1.2536
39	H	-9.6626	-3.3091	2.0128
40	H	-8.8692	-4.4071	0.8888
